# Apolipoprotein E4 and meningeal lymphatics in Alzheimer disease: a conceptual framework

**DOI:** 10.1038/s41380-020-0731-7

**Published:** 2020-04-30

**Authors:** Alexios-Fotios A. Mentis, Efthimios Dardiotis, George P. Chrousos

**Affiliations:** 1 Public Health Laboratories, Hellenic Pasteur Institute, Vas. Sofias Avenue 127, 115 21 Athens, Greece; 2 Department of Microbiology, University of Thessaly, Panepistimiou 3, Viopolis, 41 500 Larissa, Greece; 3 Department of Neurology, University of Thessaly, Panepistimiou 3, Viopolis, 41 500 Larissa, Greece; 4 University Research Institute of Maternal and Child Health and Precision Medicine, National and Kapodistrian University of Athens, Medical School, Aghia Sophia Children’s Hospital, Livadias 8, 115 27 Athens, Greece; 5 UNESCO Chair on Adolescent Health Care, Athens, Greece

**Keywords:** Genetics, Psychiatric disorders

## Abstract

The potential existence and roles of the meningeal lymphatic system in normal and pathological brain function have been a long-standing enigma. Recent evidence suggests that meningeal lymphatic vessels are present in both the mouse and human brain; in mice, they seem to play a role in clearing toxic amyloid-beta peptides, which have been connected with Alzheimer disease (AD). Here, we review the evidence linking the meningeal lymphatic system with human AD. Novel findings suggest that the recently described meningeal lymphatic vessels could be linked to, and possibly drain, the efferent paravascular glial lymphatic (glymphatic) system carrying cerebrospinal fluid, after solute and immune cell exchange with brain interstitial fluid. In so doing, the glymphatic system could contribute to the export of toxic solutes and immune cells from the brain (an exported fluid we wish to describe as glymph, similarly to lymph) to the meningeal lymphatic system; the latter, by being connected with downstream anatomic regions, carries the glymph to the conventional cervical lymphatic vessels and nodes. Thus, abnormal function in the meningeal lymphatic system could, in theory, lead to the accumulation, in the brain, of amyloid-beta, cellular debris, and inflammatory mediators, as well as immune cells, resulting in damage of the brain parenchyma and, in turn, cognitive and other neurologic dysfunctions. In addition, we provide novel insights into APOE4—the leading genetic risk factor for AD—and its relation to the meningeal lymphatic system. In this regard, we have reanalyzed previously published RNA-Seq data to show that induced pluripotent stem cells (iPSCs) carrying the *APOE4* allele (either as *APOE4* knock-in or stemming from *APOE4* patients) express lower levels of (a) genes associated with lymphatic markers, and (b) genes for which well-characterized missense mutations have been linked to peripheral lymphedema. Taking into account this evidence, we propose a new conceptual framework, according to which APOE4 could play a novel role in the premature shrinkage of meningeal lymphatic vessels (meningeal lymphosclerosis), leading to abnormal meningeal lymphatic functions (meningeal lymphedema), and, in turn, reduction in the clearance of amyloid-beta and other macromolecules and inflammatory mediators, as well as immune cells, from the brain, exacerbation of AD manifestations, and progression of the disease. Altogether, these findings and their potential interpretations may herald novel diagnostic tools and therapeutic approaches in patients with AD.

## Introduction—risk factors for Alzheimer disease (AD)

AD is among the principal causes of death and a global public health priority [1, 2]. AD is increasingly recognized as an heterogenous disease (and, as corollary, an umbrella term), which encompasses several cognitive subtypes, and whose biological and clinical manifestations may not be always co-prevalent; thus, a call for more individualized approaches has been made [3–6]. While most patients with AD manifest the late-onset, sporadic form of the disease—which may also harbor a genetic component—a small percentage (<1%) of patients carry inherited gene mutations that lead to a much earlier onset of the symptomatology [2]. Both genetic and environmental factors, reviewed in [7] and summarized in Table 1, contribute to the pathogenesis of AD. Regarding the environmental factors, we and others have shown that chronic stress might contribute to AD (reviewed also in [8–10]) via (a) sustained corticotropin-releasing hormone and cortisol effects in brain cells [11], and (b) chronic increases in systemic and brain levels of inflammatory cytokines (such as tumor necrosis factor (TNF) [12]; the latter, as components of innate immunity, are increasingly recognized for their neuroinflammatory roles in AD [13, 14].

**Table 1:** Genetic, environmental, and lifestyle risk factors for Alzheimer disease (AD) appearance or progression.

Non (purely) genetic risk factors	Genetic—chromosomal factors
Depression at any age and late-life depression^a^	*APOE4* and other gene loci, including some variants more prevalent in *APOE4*(+) patients^c,m^, and *SORL1* (neuronal apolipoprotein E receptor)^o^, and rare coding variants in apolipoprotein B^p^
Type 2 diabetes mellitus^a^	Amyloid precursor protein (inherited AD form)^d^
Frequency of social contacts—loneliness^a^	Presenilin-1 gene (inherited AD form; main cause in autosomal-dominant, early-onset AD)^d,u^
Benzodiazepines use^a^	Presenilin-2 gene (inherited AD form)^d^
Low adherence to Mediterranean diet^b,l^	Trisomy 21 (Down syndrome)^e^
Aging^g^	Immune (epigenetically regulated) response^f^ and other epigenetic events^w^
Anemia/Very low hemoglobin levels^h^, as well as very high hemoglobin levels (U-shaped relation)^x^	Variants in loci in *TREM2* and soluble TREM2 modulators, i.e., *MS4A4A* and *MS4A6A*, and loci in *CD2AP, IQCK, ACE, ADAM10, ADAMTS1, and WWOX*, and rare variants^i,j,v^ aggression
High cortical iron levels*^,s^ and plasma ferritin^t^	Variants in loci in *IGHG3, ZNF655, GPAA1, OR8G5, IGHV3-7, SLC24A3*, and lncRNA *AC099552*^q,r^
Grand multiparity^k^	Somatic brain mutations in MAPK, AMPK, and PI3K-AKT pathway^n^
Chronic stress (psychological and biological) and inflammation^g^	
Low-density lipoprotein (LDL) cholesterol^p^	
Neuropsychiatric manifestations: psychosis, aggression/agitation, affective symptoms^y^	

Sources: ^a^ [7]; ^b^ [220]; ^c^ [234]; ^d^ [235]; ^e^ [62]; ^f^ [21]; ^g^ [8]; ^h^ [236]; ^i^ [237, 238]; ^j^ [239]; ^k^ [240]; ^l^ [241]; ^m^ [242]; ^n^ [243]; ^o^ [244]; ^p^ [245]; ^q^ [246]; ^r^ [246]; ^s^ [247]; ^t^ [248]; ^u^ [249]; ^v^ [250], [251, 252]; ^w^ [253]; ^x^ [254]; ^y^ [255, 256].

*Following postmortem assessment, and mostly referring to risk for cognitive decline in already diagnosed patients with AD.

Novel insights into the etiology of AD highlight the potential contribution of the meningeal lymphatic system in the manifestations of the disease [15]. Indeed, the aforementioned connection between chronic stress, inflammation, and AD may be facilitated by the meningeal lymphatic system, as is the case in other diseases (such as multiple sclerosis (MS) [16] and post-traumatic stress disorder [17]). This could reflect the known roles of the traditional peripheral lymphatic system in inflammation and cancer, as revealed by several animal models; for instance, chronic stress in mice leads to structural changes in the lymphatic vessels of tumors and increased dissemination of metastases via transport of tumor cells through the peripheral lymphatic system [18]. Also, patients taking beta-blockers, which antagonize the endogenous stress-stimulated catecholamines at the level of the beta-adrenergic receptors, show significantly fewer proximal lymph node and distant metastases than those not receiving beta-blockers [18]; this is likely due to a reduction in catecholamine-mediated signaling events by the receptor antagonists. These findings may have broader implications for AD, granted the established link between chronic stress and neuroinflammation (reviewed in [19, 20]), as well as the known connection between neuroinflammation and AD [14, 21–24].

Here, we suggest that meningeal lymphatic-vessel function is influenced by apolipoprotein E4 (APOE4), which has been established as the leading genetic risk factor for developing AD [25]. We also present novel analyses of previously published RNA-Seq data that offer new insights into how meningeal lymphatic vessels, in association with APOE4, may contribute to the pathogenesis of AD. Our main goal is to propose a new conceptual framework on the role of reduced lymphatic function (or meningeal attenuated lymphaticness) and lymphedema in APOE4-related AD. Ultimately, in light of recent evidence, which strongly suggests that impaired brain capillary function contributes to cognitive dysfunction and AD manifestations [26], we wish to provide further evidence on the neurovascular-centered view of AD.

## The role of APOE4 as genetic risk factor for AD

**Fig. 1:**
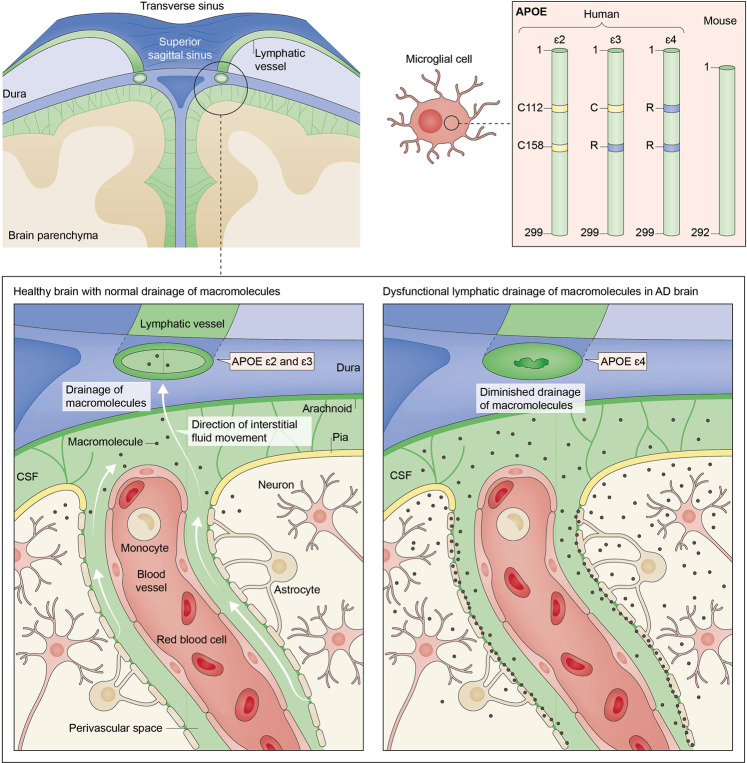
Hypothesized role of apolipoprotein E (APOE) and lymphatic vessels in Alzheimer disease (AD). Schematic depiction of mouse ApoE and human APOE isoforms (top right), with yellow indicating the presence of cysteine [C] and blue indicating the presence of arginine [R]. Illustration of brain and meningeal anatomy in a non-AD brain, showing meningeal lymphatic vessels, brain cell types, and outflow of CSF (top left and magnified in bottom left). The labeling “*APOE e4*” with an arrow pointing to the dysfunctional meningeal lymphatic vessel (bottom right) illustrates the hypothesis that APOE4 is associated with the reduction in size of meningeal lymphatic vessels, and the consequent blockade of clearance of macromolecules such as amyloid-beta. (Adapted and modified from [233] and [119], both with permission from Springer Nature).

The human *APOE* gene expresses three isoforms: *APOE2*, *APOE3*, and *APOE4*, corresponding to the APOE2, APOE3, and APOE4 proteins (Fig. 1). Although APOE has been extensively investigated for its role in liver lipoprotein metabolism (for a historical review and future perspectives, see [27]), recent data suggested that APOE is also involved in the pathophysiology of the central nervous system (CNS). For instance, *APOE*’s genetic ablation in elderly mice caused a reduction in neuritic plaques and a decrease in the associated neuronal synaptic loss and glia activation, suggesting that APOE might be helpful against certain features of aging per se [28]; however, these neuroprotective effects have not been observed in the context of the AD-associated *APP*/*PSEN1* mouse model, in which *APOE* may be neurotoxic. Interestingly, the role of APOE in the CNS is further illustrated by a recently described ultrarare mutation in the *APOE3* gene, which confers resistance to developing familial autosomal-dominant AD [29]. From a cognitive standpoint, it was previously suggested that APOE4 might increase memory in young adults; however, a recent meta-analysis failed to verify such a functional association [30].

Carrying and expressing the *APOE4*-coding allele is the chief genetic risk factor for AD, with predictive values exceeding polygenic scores for cognitive ageing in elderly populations [31, 32]. While the *APOE4* isoform is present in ~13–15% of the population, it is carried by more than 50% of individuals with late-onset AD [33]. Notably, the population attributable fraction of *APOE4* for AD (i.e., the theoretical reduction in AD incidence in the absence of the *APOE4*-coding allele) is around 7% [34]. These *APOE4* effects enhance genetic anticipation, principally in late-onset AD [35]. Of note, and according to some studies, APOE4 may be a promising therapeutic target for the disease [36].

*APOE4* status is also linked to dementia with Lewy body disease, with decreased levels of *APOE* methylation having also been implicated in this disease [37–39]. *APOE4* status has also been linked to Parkinson disease-related dementia [40], MS, tauopathy, vascular dementia (whose partial genetic overlap with AD may be explained by *APOE* genetic status) [41], mixed vascular dementia and AD [42], chronic traumatic brain injury [43], cerebral amyloid angiopathy [44], and cerebrovascular disease [45], as well as positive status of transactive response DNA-binding protein 43 (a protein previously linked to AD, frontotemporal dementia, and amyotrophic lateral sclerosis) [46]. While *APOE2* status can postpone age-at-onset of AD [47], an individual possessing a single *APOE4* allele has a threefold increased risk for AD late in life, and carrying two alleles has been associated with a higher than tenfold risk [48]. Of note, age, region, and ethnicity may partially modulate this association [49–52]; for instance, Hispanic carriers of *APOE4* seem to have a higher amyloid load than non-Hispanic carriers [53].

Mechanistically, diploidy for human *APOE4* is thought to contribute to the aggregation of amyloid-beta in the brain (for comprehensive reviews, see [25, 44, 54]), possibly through the neuronal receptor LRP1 [55]. Further experimental evidence supporting a role of APOE4 in amyloid-beta aggregation came from studies using Pittsburgh Compound-B-positron emission tomography (PET) imaging [56]. In one of these studies, *APOE4* status was associated with accelerated cognitive dysfunction in individuals whose PET results were positive for amyloid-beta [57]. From a biochemical point of view, amyloid-beta fibrils were more strongly associated with APOE4 than with APOE3 or APOE2 [58]. Conversely, amyloid-beta protofibrils had higher stability in their association with APOE3 or APOE2 than with APOE4 [59].

Patients with sporadic AD may exhibit impairments in amyloid-beta clearance, without major changes in the de novo production of the peptide [60]. In mice, APOE4 directly disrupts clearance of amyloid-beta across the blood–brain barrier (BBB), suggesting that impairment in the neurovascular function of the BBB may contribute to AD etiology. To determine the critical stage of amyloid fibril (seeding stage) and plaque (plaque stage) formation, in which APOE4 exerts its strongest effect, Liu et al. developed a cell-type specific, Cre-floxed-mediated inducible mouse model to control expression of astrocytic *APOE4* during amyloid fibril and plaque formation [61]. Their data indicated that APOE4 had its greatest impact during the seeding stage of amyloid-beta formation, probably by impeding amyloid-beta clearance and promoting its aggregation [61]. Interestingly, these associations may share similarities to those observed in brain pathologies in Down syndrome (trisomy 21) patients. These patients carry an extra copy of chromosome 21, in which the amyloid precursor protein (APP) is located. Trisomy 21 results in APP overexpression [62]; however, others studies support that trisomy 21-related dementia may be caused by overexpression of non-APP genes leading to a decrease in the soluble amyloid-beta-38, and amyloid-beta-40 [63]. Over two thirds of older adults with trisomy 21 die from dementia; among them, the risk of premature death is increased by almost sevenfold in *APOE4* isoform carriers [64]. Lastly, the modulatory role of APOE4 on amyloid-beta formation has been partly attributed to decreased serum and brain concentrations of APOE in *APOE4* carriers vs. non-*APOE4* carriers, and not to the *APOE4* allele(s) per se [44].

AD has been associated with tauopathy, another proteinopathy characterized by the pathological accumulation of tau protein in the brain (for a review on tau’s role in physiology, see [65]). The link between tauopathy and APOE4 has been addressed in several studies. In tau-expressing transgenic mice, APOE4 exacerbated tau-mediated neurodegeneration, causing increases in brain atrophy and neuroinflammation, and alterations in glial cell function; this effect was seemingly independent of amyloid-beta pathology [66]. In addition, APOE4 increased levels of phosphorylated tau and its extracellular release by neurons, a process independent of glia cells [67].

APOE4 may also contribute to AD symptomatology via mechanisms that are not related to either amyloid-beta or tau. First, APOE4 causes a decrease in the levels of exosomes released in the brain interstitial space, together with a reduction in endosome/exosome pathway-related gene expression [68]. These phenomena are linked to malfunction of the lysosomal system and the impaired degradation of cellular debris, which, in turn, may lead to accumulation of amyloid-beta, and hence, to neurodegeneration [68]. Second, the presence of APOE4 hinders neuronal responsiveness to reelin, a glycoprotein that controls neuronal migration and synaptic transmission, while it promotes thrombosis and hemostasis [69]. In doing so, APOE4 may reduce, in patients with AD, the protective effects of reelin against beta-amyloid-induced cognitive impairment [70]. Third, APOE4 may lead to dysfunction and eventual death of gamma-aminobutyric acid-expressing interneurons in the hippocampus, a brain region that is severely impaired in AD [71]. Because these neurons are inhibitory, their absence causes hyperexcitability of the entire hippocampal network, leading to the impairments observed in AD [72] (for this conceptual framework, see [73]). Finally, APOE4’s role in AD may involve a dysfunctional immune system [74]. Indeed, activation of the innate immune response is considered a disease-promoting factor in AD [75], and APOE4 regulates different aspects of the inflammatory reaction (e.g., microglia activation) [76].

In particular, one of APOE4’s effects on innate immunity appears to be, at least partly, via the triggering receptor expressed on myeloid cells 2 (TREM2), which is primarily expressed in the CNS microglia [66] and involved in microglial disorders (microgliopathies) [77]. Indeed, TREM2 is involved in APOE4’s downstream activation of microglia [78, 79]. TREM2 double knockout mice exhibit increased amyloid plaque formation with less plaque-associated APOE [80]. Importantly, genome-wide association studies have corroborated the crucial roles of TREM2 and other microglia-associated proteins, such as PLCG2 and ABI3, in AD pathophysiology [81–86]. This evidence points to a delicate interaction between APOE (including the APOE4 isoform), TREM2, and amyloid-beta. Further research will determine whether this interaction plays a key role in the pathophysiology of human AD.

## APOE4 in AD-associated neurovascular and cerebrovascular function

Recent advances in our understanding of AD highlight the role of neurovascular function in the pathogenesis of AD, including cognitive impairment, thus warranting further investigation of vascular markers of the disease [87]. Studies linking AD and neurovascular function have focused on fibrinogen [88], imaging biomarkers of amyloid-beta and neurodegeneration (e.g., plasma neuronal-enriched extracellular vesicles), MRI, amyloid PET, tau PET, and fluorodeoxyglucose PET (reviewed in [89–92]). Of particular interest is the role of the neurovascular system in clearing amyloid-beta from the brain. For example, Nation et al. [93] detected hippocampal capillary damage and BBB breakdown in people with early cognitive dysfunction, independently of amyloid-beta and tau pathologies; these findings suggested that BBB dysfunction can serve as an early biomarker of human cognitive dysfunction, a precursor of AD [94].

Notably, APOE4 itself has been linked to impaired BBB function. In transgenic mice, expression of *APOE4* (but not other *APOE* variants) leads to breakdown of the BBB; this phenomenon is mediated by the activation of a proinflammatory pathway in pericytes (i.e., perivascular cells) [95]. BBB breakdown leads to passage of neurotoxic proteins and other substances in the brain parenchyma, as well as decreased blood flow in the brain microvasculature. These events likely occur prior to neuronal dysfunction, and they may lead to neurodegenerative changes [95]. In the clinic, similar observations on BBB breakdown have been inferred from increases in cerebrospinal fluid (CSF)/plasma albumin quotients in *APOE4* carriers [96].

Based on neuroimaging data, cerebrovascular diseases can increase the risk for AD and tau pathology [97–99]. High levels of low-density lipoprotein (LDL), also associated with vascular diseases, may lead to amyloid accumulation in *APOE4* carriers [100]. Moreover, cardiovascular risk factors and type 2 diabetes mellitus also increase the risk of dementia, suggesting that impaired metabolism also contributes to AD [101]. Experiments in cells and animal models have shed light on some putative mechanisms. For instance, APOE and circulating high-density lipoprotein-mediated amyloid-beta transport in bioengineered human cerebral vessels, with APOE4 being less effective than APOE2 in this regard [102]. Also, exogenously administered amyloid-beta was more effectively cleared through the BBB in mice overexpressing the low-density lipoprotein receptor [103]. Taking this further, the loss of mouse *ApoE* in pericytes was shown to inhibit the clearance of aggregated amyloid-beta-42 on multispot glass slides; this inhibition was rescued by human APOE3 but not APOE4 [104]. Collectively, the above findings partly explained APOE4’s pathogenic role and provided further insights into the neurovascular-centered view of AD.

## APOE4 and the meningeal vs. traditional peripheral lymphatic system

Considering the strong interactions between the vascular and peripheral lymphatic systems, as well as the shared features between the meningeal and traditional peripheral lymphatic systems, it is worth exploring the potential roles of APOE4 in the meningeal lymphatic system, following insights from the traditional peripheral lymphatic system as well.

### The traditional peripheral vs. meningeal lymphatic system

The peripheral lymphatic system plays a crucial role in the clearance of many substances, such as cellular debris, proteins, lipids, and other macromolecules from peripheral organs. Thus, impaired clearance of various interstitial space macromolecules has been often linked to abnormalities in peripheral lymphatic vessels, such as enlargement of these vessels and leakage of their contents into the surrounding space (for reviews on this field, see [105, 106]). According to the predominant theories, peripheral lymphatic vessels originate in early development from the venous endothelial system by forming lymphatic endothelial cell progenitors. However, according to recent lineage tracing studies, progenitors of peripheral lymphatic endothelial cells may also include dermal blood capillaries, lymphangioblasts, blood cell progenitors, hemogenic endothelial cells, and organ-specific (e.g., heart) endothelial cells. These progenitors, at least in animal models, may also include venous endothelial cells outside the heart, and lymphatic progenitors stemming from the blood-producing yolk sac endothelium. Progenitor cells derived from the yolk sac may be particularly relevant in the cardiac lymphatic vasculature (for all above, see [108–109]).

Until recently, it was believed that the meninges lacked lymphatic vessels, principally because of the dogmatic view on the immuno-privileged status of the brain. The Italian scientist Paolo Mascagni first described the meningeal lymphatic system in the 1800’s, but his conclusions were disputed until recently (for works spanning all this period, see [111–115–116]). In addition, despite description of Mendelian disorders with peripheral lymphedema and cognitive impairment (e.g., in Hennekan syndrome [117]) or nonspecific neurological signs (e.g., generalized lymphedema associated with neurologic signs syndrome [118]), the existence of meningeal lymphatics remained disputed. Therefore, until some years ago, it was thought that meningeal lymphatic vessels did not exist, and let alone play any role in CNS physiology or pathophysiology.

Intriguingly, several studies have now demonstrated that lymphatic vessels exist within the meninges of the CNS [120–121] (reviewed in [122, 123]). This is a rediscovery, one heralding a delayed history [124]. Alongside with the confirmatory findings suggesting that Schlemm’s canal in the eyes had features resembling lymphatics that were evolutionarily conserved [125], this rediscovery suggests inter alia the notion that investing on mining of hidden pearls can be crucial in advancing biomedical research. Fascinatingly, even peripheral lymphatic vessels were also rediscovered after the initial Hippocrates and Erasistratus of Ceos analyses of white blood-filled vessels, and mesenteric milky arteries, respectively, till the 17th century Aselli’s observation of a dog’s milky veins [126] (mentioned in [127, 128]).

These initial descriptions of meningeal lymphatic vessels were followed by further studies in humans. In particular, by coupling MRI findings with postmortem analyses, researchers demonstrated that meningeal lymphatic vessels existed alongside blood vessels in humans and nonhuman primates [129]. More recently, meningeal lymphatic vessels in the basal part of the mouse skull were shown to support the drainage of macromolecules (metabolites, debris, and immune cells) from the CSF. This most likely follows exchange of solutes, debris, and immune cells with the brain interstitial fluid (ISF), via the efferent paravascular glial lymphatic (glymphatic) system into the meningeal lymphatic vessels, ultimately draining into the conventional cervical lymphatic system, including local lymph nodes [15, 119, 121] (for a review, see [130]).

Drainage of glymphatic fluid through the newly described basal meningeal lymphatic vessels in the mouse skull is quite efficient [131]. This higher draining capacity of the basal than the dorsal meningeal lymphatic vessels may be partly explained by their respective proximity to the subarachnoid space, and by their location, which is independent of nerve fibers. These meningeal lymphatic vessels are presumably distinct from the traditional peripheral lymphatic system [131]. Notably, the function of the basal meningeal lymphatic vessels was compromised in ageing mice [131]. Mechanistically, lymphatic wall hyperplasia may take place in response to increased capillary lymphatic pressure, as it occurs in peripheral lymphedema. The latter has been linked to aberrant type IV collagen distribution, a reduction in lymphatic valves, potentially via decreased Prox*1* and *Foxc2* expression [131]. Moreover, lymphatic endothelial cells in aged mice showed significantly altered intercellular junction types, along with reduced functionality for glymphatic drainage through the basal meningeal lymphatic vessels [131]. Altogether, these findings suggest that aged basal meningeal lymphatic vessels are associated with reduced lymph flow; however, to our knowledge, whether similar pathology is present in human AD has not been investigated as yet.

Although not directly applicable to human pathophysiology, valuable lessons can be learned from observations in animal models, such as the zebrafish. In the latter, mural lymphatic endothelial cells produce vascular growth factors and promote accumulation of LDLs from the bloodstream [120, 132]. As noted above, meningeal lymphatic cells are derived from the blood vasculature and in zebrafish, this phenomenon is mediated by the *Vegfc*–*Vegfd*–*Ccbe1*–*Vegfr3* pathway; this suggests that these cells are probably lymphatic capillary-type endothelial cells [132]. Ablation of meningeal lymphatic vessels in mice using a photodynamic drug, Visudyne (verteporfin), resulted in (a) reduced drainage of macromolecules, and (b) impairments in cognitive performance. Altogether, these findings point to a major role of meningeal lymphatic vessels as a conduit in the clearance of macromolecules from the mouse brain. These macromolecules most likely reach the meningeal lymphatic system through the efferent glymphatic drainage system formed by astrocytes around brain blood vessels, as described in [133]. The functions of both the glymphatic and meningeal lymphatic systems are compromised during ageing [15, 134, 135].

In summary, the studies discussed above suggest that the meningeal lymphatic vessels can be, at least in part, responsible for the dysfunctional clearance of macromolecules from the brain. Here, we theorize that this process could contribute to the decline of cognitive function associated with age and neurodegenerative diseases, such as AD [15].

### Crosstalk of meningeal and cervical lymphatic vessels

The (re)discovery of meningeal lymphatic vessels is now an acknowledged paradigm shift. However, the presence of meningeal lymphatic vessels should not be considered in isolation but rather in the context of the conventional lymphatic system, including the most proximal cervical lymph vessels and nodes (CLNs). CLNs are already known to play a major role in MS and other neuroinflammatory diseases [136], in which brain-peripheral immune crosstalk has been observed [137] (potentially because of the presence of autoantigens or microbial risk factors [138, 139]). The role of the CLNs in AD, however, has been investigated far less. Indeed, although follicular dendritic cells (FDCs) within the CLNs develop from perivascular precursors [140], their potential links to meningeal lymphatic vessels or to AD are largely unknown.

To our knowledge, FDCs have been mostly linked to the prolonged retention of human immunodeficiency virus (HIV)-1/simian immunodeficiency virus infection in the context of CLNs [141], through a number of mechanisms, including cycling endosomes [142]. Taken further, FDCs seem to indirectly contribute to transmitting the virus to CD4^+^ T follicular helper cells; this is performed in a B-cell-mediated manner [143]. Elucidating how meningeal lymphatic vessels interact with CLN-associated FDCs could shed light on their crosstalk in MS and, notably, AD. Interestingly, a very recent single-cell transcriptomics study reported *APOE* among the genes that are differentially expressed in mouse CLNS [144], thus suggesting a role of this gene (and, as corollary, of its variants such as *APOE4*) in CLNs-related pathological processes.

In the next section, we summarize the evidence linking APOE4 to the meningeal lymphatic system, and how this may contribute to the pathophysiology of AD.

### Investigating the potential links of APOE4 and the lymphatic system

Brain tissue samples from patients with AD compared with normal brains show several microvascular alterations, with morphological studies reporting fusiform dilations, tortuosities, and abnormal branching, an overall decrease in the density of capillaries, mitochondrial abnormalities in capillary endothelial cells, and degeneration of pericytes [145]. These studies are aligned with previous data, showing focal constriction of many terminal arterioles and irregularly shaped smooth muscle cells, and capillaries with both abnormal constrictions and dilatations, in patients with AD [146].

In addition, data from both biopsies of patients with AD and mouse AD models have provided some insights into the mechanisms underlying capillary constriction and reduced blood flow; in this regard, amyloid-beta enhanced oxidative stress in pericytes via NADPH oxidase-4, leading to endothelin-1-mediated effects of endothelin A receptors on capillary-related pericytes [26]. In line with these observations, clinical studies suggested that blood pressure-lowering drugs, such as calcium channel blockers (which lower arterioral rather than venous resistance [147]) effectively increased cerebral blood flow in patients with AD [148]. To our knowledge, the effects of selective endothelin receptor A antagonists in cerebral blood flow in AD patients have not been investigated.

The extent to which the meningeal lymphatic system of patients with AD exhibits the aforementioned abnormalities, and how APOE4 affects meningeal lymphatic vessels, are both worthy of further investigation. Characterizing the branching morphology of these vessels to detect any pathological remodeling in APOE4-related AD could provide novel insights into the pathophysiology of the disease. In this context, Lim et al. reported that *ApoE*-deficient (*ApoE*−/−) mice exhibited distinct lymphatic phenotypes, including tissue swelling, leaky peripheral lymphatic vessels, a significant dilatation of capillary peripheral lymphatic vessels, and a reduction in the transport of lymphatic fluid and dendritic cells from peripheral tissues [149]. Moreover, peripheral lymphatic vessels reduced their recruitment of smooth muscle cells and showed an altered distribution of the lymphatic endothelial hyaluronic acid receptor 1 (LYVE-1) [149].

It should be noted, however, that meningeal and peripheral lymphatic vessels have different characteristics. Of note, the meningeal lymphatic vessels are less complex, and show smaller lymphatic branching and fewer valves to prevent backflow of lymph [121]; nonetheless, basal meningeal lymphatic vessels have more clearly defined valves than dorsal meningeal lymphatic vessels [131]. Interestingly, the metabolic pathways mediating cholesterol homeostasis in the brain also differ from those of peripheral tissues. The BBB prevents peripheral cholesterol from entering the brain, in which cholesterol is largely synthesized by astrocytes and oligodendrocytes [150].

Omnipresent endothelial cells and peripheral lymphatic vessels may share some ontogenetic features. Specifically, they may share common embryonic cellular origins; thus, peripheral lymphatic vessels may be reprogrammed to become blood vessels in case of blood flow-related events, such as shear stress [151]. Given these close ontogenetic similarities between blood and peripheral lymphatic vessels, and APOE’s established role in the peripheral lymphatic system [145, 149], it would be intriguing to decipher whether (a) meningeal lymphatic vessels play a role in amyloid-beta clearance, and (b) amyloid-beta clearance is compromised by the *APOE4* isoform (analogous to the *APOE4* isoform’s effects on the BBB and meningeal lymphatic disruption [152]). In this regard, a recently published study showed that ablation of the meningeal lymphatic vessels in the 5xFAD mouse model of AD resulted in a striking deposition of amyloid-beta in the meninges, macrophage recruitment to large amyloid-beta aggregates, and an increase in the amyloid-beta plaque load in the hippocampus [15]. Moreover, as compared with healthy control mice, 5xFAD mice showed vascular amyloid-beta pathology in the cortical leptomeninges and beta-amyloid depositions in the dura mater adjacent to the superior sagittal sinus [15]. Together, these findings underscore the crucial roles of CSF and ISF fluid drainage through meningeal lymphatic vessels for normal brain physiology and pathophysiology.

In addition, the worsening of amyloid-beta pathology upon disruption of the meningeal lymphatic system in mouse models of AD suggests that dysfunction of these vessels exacerbates AD pathology. Importantly, the AD transgenic mouse models (J20 and 5xFAD) used in [15] showed no differences from controls in their meningeal lymphatics (for review, see [130]). These mouse models, however, are probably less relevant to APOE4-induced AD, given that they are driven by overexpression of mutated human APP transgenes. Notably, the 5xFAD model presents a more aggressive phenotype due to the expression of mutated presenilin (PS1) in the same construct [153]. Hence, these models may be better suited for early-onset AD, whereas the involvement of APOE4 may be more relevant to late-onset AD; thus, these results may not capture the effects of APOE4 as a major AD risk factor on meningeal lymphatic vessels.

## APOE4, aquaporin 4, and the glymphatic vs. the meningeal lymphatic systems

We contend herein that the efferent glymphatic system of paravascular astroglial channels draining into the meningeal lymphatic system could also be involved in AD pathogenesis. The glymphatic system supports CSF–ISF exchange and clearance of interstitial waste from the brain parenchyma [133], not only in mice, but also in humans [154]. Moreover, the glymphatic and, as corollary, the meningeal lymphatic system (for a discussion on their interconnection and a schematic representation of their differences, see [155]) could potentially play a role in the clearance of metabolites, macromolecules, such as amyloid-beta, and inflammatory mediators, as well as immune cells. The glymphatic system’s physiology may be affected by the natural arterial pulsations of solute exchange in the CSF–ISF interphase, with major increases of this exchange taking place in the brain during deep sleep [156, 157].

In mice, disruption of the astroglial aquaporin 4 (AQP4) water channel (which regulates the glymphatic clearance of macromolecules [158]) resulted in the accumulation of amyloid-beta in the hippocampus after blockade of meningeal lymphatic drainage via ligation of deep cervical lymphatic nodes (LdcLNs) [159]. Mice with deficits in both glymphatic and meningeal lymphatic clearance exhibited increased microglial activity and activation of the microglial inflammasome, as well as enhanced hippocampal neural apoptosis and reduction of cognitive function [159]. Moreover, tau levels were increased in the LdcLNs mice, but not in the *Aqp4*-null mice [159].

The glymphatic system contributes to the transport of lentiviral-delivered APOE3 to neurons [152]. Thus, in addition to its clearance role, the glymphatic system may help distribute essential molecules throughout the brain (although the brain retention of APOE3 or APOE2 is lower than that of APOE4) [152]. However, this notion is still in debate; for example, concerns have been raised on the role of AQP4 in the convective transport of solutes produced by the CSF–ISF exchange, calling for a reappraisal of the notion of a glymphatic system [160]. In addition, there is evidence supporting a more diffusive mode of transport that does not involve AQP4 [161]. Nevertheless, a recent thorough investigation and meta-analysis suggested that AQP4 dysfunction could impair CSF influx (and, as a possible corollary, the exchange of solutes and cells with the ISF) [162]. Therefore, further studies are needed to help us understand how the glymphatic and meningeal lymphatic systems are interconnected with regards to AD pathology, and how APOE4 influences this crosstalk.

## Expression of lymphatic-vessel genes in *APOE4*-expressing cell types of the brain

### Cell-specific effects of APOE4 in AD pathology

The cell-specific effects of APOE4 in AD pathology are poorly understood. Lin et al. attempted to determine which cell types in the human brain are affected by the expression of the *APOE4* isoform. To this end, they applied CRISPR/Cas9 gene editing to create *APOE4* knock-in human iPSCs stemming from a single iPSC line of a human subject without AD; all these modifications were conducted in an otherwise isogenic background [163]. Using a differentiation system for iPSCs, they generated various brain cell types and compared gene expression resulting from the *APOE4* knock-in to that of an analogous *APOE3* knock-in [163]. They found that the expression of hundreds of genes was altered in iPSC-derived neurons, astrocytes, and microglia; many of these were also aberrantly expressed in postmortem samples of patients with AD [163]. The observed cellular defects caused by *APOE4* expression included (a) an increased number of synapses and elevated amyloid-beta-42 secretion in neurons, (b) defects in amyloid-beta uptake and cholesterol accumulation in astrocytes, and (c) an aberrant morphology correlating with reduced amyloid-beta phagocytosis in microglia [163]. Therefore, by harnessing gene editing approaches in an otherwise isogenic background, this study has the potential to offer interesting mechanistic insights concerning APOE4.

To this end, we theorize below that specific cell types could be used as surrogates for lymphatic vessels to provide initial clues for our conceptual framework.

### The common mesodermal origin of lymphatic-vessel cells and microglia

To provide a rationale for the use of surrogates to understand meningeal lymphatic-vessel functions, we attempt here to highlight the common mesodermal origin of lymphatic vessels and microglia. On the one hand, the ontogenetic origins of microglia cells, now shown to comprise at least nine transcriptionally distinct subtypes [164], have remained controversial. Experimental evidence suggested that microglial homeostasis in the adult brain was not mediated by postnatal hematopoietic progenitors, whereas microglia development in mice required the colony stimulating factor-1 receptor. Based on an in vivo lineage tracing study, the origins of adult microglia were attributed to primitive myeloid progenitors, which, in turn, originated from the yolk sac (i.e., an element that arises before mouse embryonic day 8, and that is associated with the splanchnic layer of the lateral plate mesoderm [165]). On the other hand, the ontogenetic origin of lymphatic vessels has been extensively discussed. Some authors suggested that the lymphatic vessels emerge from the primary lymph sacs, which develop from primitive veins. Others suggested that they first arise from mesenchymal spaces, and then connect to the primary sacs, thereby, indirectly, to the venous system [127]. However, in both sides of the argument, a common mesodermal origin has been proposed for microglia and lymphatic vessels. The above suggests that microglial cells might be used as a proxy to study lymphatic cells, particularly in the context of an iPSC line. Potente and Mäkinen [166] have summarized the evidence on peripheral lymphatic-vessel origin, noting (a) the undefined, in many cases, origin of nonvenous lymphatic endothelial cells, (b) the migration of lymphatic endothelial cells from the lymphatic vasculature, and (c) the fact that blood-forming endothelial progenitors undergo vasculogenesis to form lymphatic vessels.

### Reanalysis of data from previous studies

Considering the above information, we theorize that the cell-type specific RNA-Seq dataset from Lin et al. could allow us to examine, at the level of gene expression, the hypothesis that meningeal lymphatic vessels are affected by APOE4 [163]. Encouraged by both recent calls to make use of open public genomic data [167] and the notion that iPSCs can be an attractive way to model AD pathology [168], we reanalyzed the data from the study of Lin et al. deposited in GEO (GSE102956). Our goal was to identify genes that were differentially expressed in the *APOE4* (pathological state) vs. the *APOE3* (control) knock-in. We were interested in studying the expression of selected gene markers in parental cells in the starting iPSCs and iPSCs-derived neurons, astrocytes, and microglia (see the “Appendix” section for the methods applied). These selected gene markers referred to lymphatic-vessel-related genes or/and genes in which well-characterized missense mutations have been linked to peripheral lymphedema (Table 2).

**Table 2:** Markers of human lymphatic vessels.

Lymphatic markers and lymphatic-related markers	
Prox1	Podoplanin (PDPN)
CD31 (PECAM1)	VEGFR-3
LYVE-1	CCL21
CD68 negativity^a^	CD45 (PTPRC)^b^
Initiation of valve formation	
GATA2	Ephrin type B2 (EFNB2)
FOXC2	Prox1
Glymphatic and astrocytic markers	
Aquaporin 4	
Lymphatic valve maturation	
Integrin alpha-9 (ITGA9)	Fibronectin 1 (FN1)
Connexin 43 (GJA1)^c^	
Lymphedema	
VEGF-C^d^	VEGFR-3 (or FLT4)^e^
FOXC2^f^	GATA2^e^
CCBE1^e^	GJC2^e^

^a^According to [119], the absence of CD68 positivity on Lyve-1 positive structures has been noted.

^b^According to [257], a striking colocalization of CD45(+) leukocytes with the developing lymphatics has been noted in mice.

^c^According to [258], astrocytes lacking GJA1 showed reduced Apoe protein levels as well as impaired amyloid-beta phagocytosis.

^d^VEGF-C exposure increases the diameter of meningeal lymphatic vessels; the latter completely fail to develop upon VEGF-C and VEGFD inhibition.

^e^Mutant gene forms, with functional, missense mutations linked to lymphedema.

^f^Marker of initiation of valve formation, and mutant gene forms linked to lymphedema-distichiasis syndrome.

Source: Gene markers based on previous reports from [60, 119, 131, 257, 259–265] and Online Mendelian Inheritance for Man Database (www.omim.org) (Accessed January 2019).

**Fig. 2:**
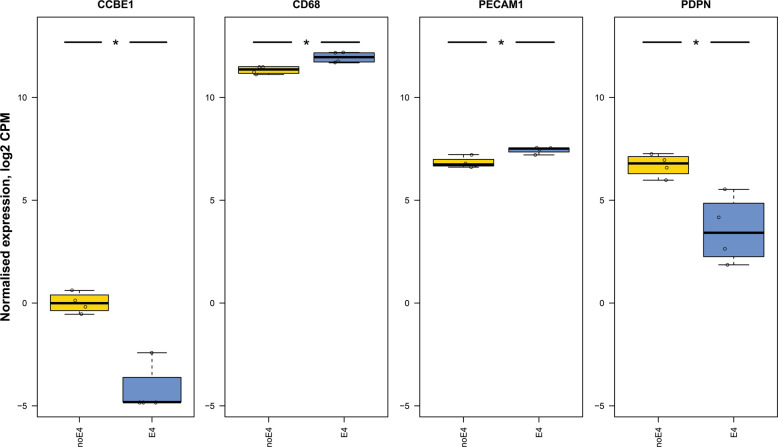
Box and whisker plots showing meningeal lymphatic marker expression levels in iPSC-derived *APOE4* and *APOE3* (control) knock-in cells, with iPSCs being derived from an unaffected subject carrying *APOE3* alleles, in which cells were gene edited using CRISPR/Cas9 to generate *APOE4* iPSCs from parental *APOE3* cells, and isogenic iPSCs were then differentiated into neurons, astrocytes, and microglia-like cells. Normalized gene expression of meningeal lymphatic markers and related genes in cells expressing knock-in of either non-*APOE4* (blue) or *APOE4* (yellow). Differentially expressed genes are marked with an asterisk to indicate statistical significance: asterisk (*) for false discovery rate (FDR) ≤ 0.05; double asterisks (**) for FDR ≤ 0.01; and triple asterisks (***) for FDR ≤ 0.001. The perpendicular bars represent the standard deviation (SD). Based on reanalysis of data from the published source in [163]. Please note that the scale of the graph may differ between the different depicted genes.

Using the available technical replicates derived from these iPSCs, our reanalysis did not reveal statistically significant differences in the expression of genes of interest in iPSCs, neurons, and astrocytes (data not shown); this was based on searches on all gene markers presented in Table 2. It should be noted, however, that some of these gene markers may not be uniquely expressed in lymphatic endothelial cells. Interestingly, in the microglial cells, we found statistically significant differences between the *APOE4* and *APOE3* knock-in cells in the expression of several genes related to features of lymphatic vessels (Fig. 2). In the *APOE4* knock-in cells, we detected significantly decreased levels of expression of *CCBE1* (a marker linked to lymphedema) and *PDPN* (adjusted *p* values ≤ 0.05, in both cases), and significantly increased levels of *CD68* (a marker whose absence of detection during immunohistochemistry staining is linked to features of lymphatic vessels), as well as, *PECAM1*, compared with the *APOE3* parental cells (adjusted *p* value ≤ 0.05, in both cases). Collectively, except for *PECAM1*, these findings indicate a more pronounced presence of RNA transcripts corresponding to lymphatic or lymphatic-related markers in cells expressing *APOE3* than in cells expressing *APOE4* (Fig. 2).

**Fig. 3:**
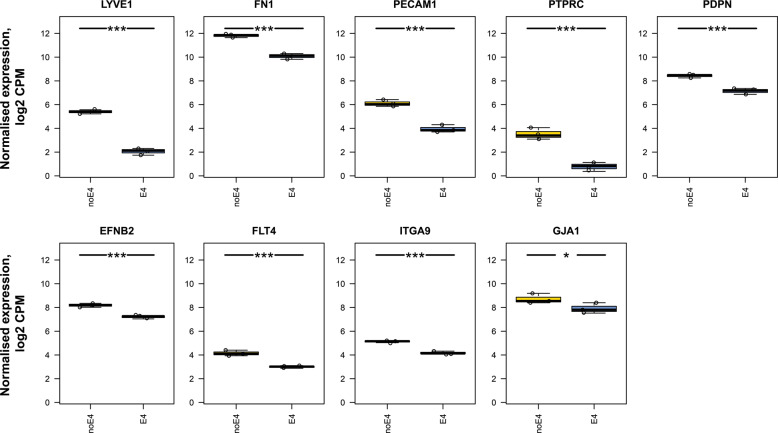
Box and whisker plots showing meningeal lymphatic marker expression levels in iPSCs derived from individuals with sporadic AD *APOE4* (e4/e4) genotype, with parental iPSCs lines edited to E3 (e3/e3) genotype, and cerebral organoids generated from each of the lines. Normalized gene expression of meningeal lymphatic markers and related genes in cells expressing knock-in of either non*-APOE4* (blue) or *APOE4* (yellow). Differentially expressed genes are marked with an asterisk to indicate statistical significance: asterisk (*) for false discovery rate (FDR) ≤ 0.05; double asterisks (**) for FDR ≤ 0.01; and triple asterisks (***) for FDR ≤ 0.001. The perpendicular bars represent the standard deviation (SD). Based on reanalysis of data from the published source in [211]. Please note that the scale of the graph may differ between the different depicted genes.

To avoid the possibility of selective reporting bias in our reanalysis, we performed a systematic query to identify other studies in the GEO database focusing on human-derived samples and APOE (Appendix). Twenty-two GEO datasets were identified in total, including the one described above (GSE102956). Fourteen datasets were excluded for various reasons (for the reasons of exclusion, see Supplementary File 1). A total of eight datasets were ultimately considered, including GSE102956 described above (Supplementary File 1). Of interest, we found an additional in vitro study [169] (GSE117588) in which iPSCs were derived from individuals with sporadic AD who had the *APOE4* (e4/e4) genotype. Parental iPSC lines were then edited to *APOE3* (e3/e3) genotype, and cerebral organoids (with neural progenitor cells) were generated from each of the lines; arguably, this is more relevant to AD pathology. Using this dataset, we identified significant differences in the differential expression of a range of genes related to lymphatic cells in the *APOE4* vs. the non-*APOE4* cells. Specifically, we found that *APOE4* cells of the cerebral organoids showed significant decreases in the expression levels of *EFNB2*, VEGFR-3 *(FLT4)*, *FN1*, *ITGA9*, *LYVE1*, *PDPN*, *PECAM1*, and *PTPRC* (all with adjusted *p* values ≤ 0.001), as well as, *GJA1* (adjusted *p* value ≤ 0.05) (Fig. 3). We observed, though, a significant increase in the housekeeping gene glyceraldehyde 3-phosphate dehydrogenase (data not shown) in the non-*APOE4* cells, which might raise concerns on the degree of validity in the current analysis. However, some authors have expressed concerns about accounting for housekeeping genes in genome-wide RNA-Seq analyses, thus suggesting global normalizations rather than normalizations based on housekeeping genes (for a discussion on this issue, see [170]).

In parallel to this, we did not find significant differences in the differential expression of genes of interest in studies from human tissues, such as in (GSE48350) [171] (Supplementary Fig. 1), in (GSE106241) [172] (Supplementary Fig. 2), or in (GSE125050) [173] (Supplementary Fig. 3) aside from the *PDPN* gene in (GSE29652) [174] (Supplementary Fig. 4). This could indicate that, contrary to the isogenic conditions of in vitro studies on *APOE4* vs. *APOE3*, other parameters (e.g., stage of disease, gender, and age) could have decreased the power of analysis by adding significant non-APOE-relevant variation. Finally, lack of replication in the SAGE-based study (GSE6677) [175] (Supplementary Fig. 5) precluded a statistical analysis of the data (for further details on the reanalyzed studies, see Supplementary File 2).

Although the above studies are limited in their ability to predict true anatomical effects, a logical extension of our initial results is that the *APOE3*-expressing cells exhibit more prevalent characteristics of lymphatic cells than *APOE4*-expressing cells. Furthermore, in *APOE4*-expressing cells, we observed reduced expression of gene markers linked to peripheral lymphedema (e.g., *VEGFR3* (*FLT4*) [176]) or lymphatic valve formation (e.g., *FN1* [177], *GJA1* [178, 179], and *ITGA9* [180]). Together, these findings indicate a deficient effect of *APOE4* on certain, iPSCs-derived, lymphatics-related functions.

### Assessing the relevance of reanalyzed data on CNS lymphatic biology

Could these insights, which are based on transcriptomic data obtained from iPSCs, be relevant to CNS lymphatics biology? Extensive experimental research on the effects of APOE4 on peripheral or CNS lymphatic endothelial cells/vessels is required before drawing any certain conclusion. With regard to the first set of data (Fig. 2), a major consideration was that microglia should not express *AQP4* (classical marker of astrocyte endfeet in the brain) or *PECAM1* (classical marker of brain vascular endothelial cell). This is because, at the single-cell level, the transcripts for *PECAM1* in vascular endothelial cells (either of blood or lymphatic origin) and for *AQP4* in astrocytes are a hundred, if not a thousand, times higher than in microglia. This consideration could hinder the interpretation of the results regarding iPSC-derived microglia.

Another issue, in the studies that we reanalyzed, was the ontogenetic stage at which the *APOE4*-knock-in iPSC-derived cells started to express *PECAM1*, *PDPN*, *CCL21*, *LYVE-1*, *FLT4*, and *PROX1*, at similar transcript levels to lymphatic endothelial cells. This is a relevant issue because the above *APOE4*-knock-in cells may not directly reflect the physiology of lymphatic endothelial cells.

### The notions of attenuated lymphaticness, meningeal lymphedema, and lymphosclerosis in APOE4-related AD

Despite these considerations, the preliminary findings of our reanalyses, regardless of the origin of iPSCs, point toward higher expression of RNA transcripts corresponding to lymphatic or lymphatic-related markers in *APOE3* vs. *APOE4* cells. Thus, despite the distinct genetic signature and function of meningeal lymphatic vessels, our data suggest that APOE4-related AD may be linked to attenuated lymphatic features, such as shrinkage of meningeal lymphatic vessels, weakened function of meningeal lymphatic valves, and, in turn, reduced lymphatic flow. Taking this further, and in line with previous findings on ageing in rodents [131], we propose to label these APOE4-mediated cellular events with the term attenuated lymphaticness. Accordingly, if meningeal lymphatic vessels undergo alterations resembling lymphosclerosis (as previously defined in [181, 182]), the CSF after the exchange of solutes and cells with ISF coming through the efferent glymphatic system (a term we wish to describe as glymph) could be obstructed in terms of their flow, causing local stagnation (i.e., meningeal lymphedema). Whether or not this meningeal lymphedema could affect the removal of cellular debris, amyloid-beta, and tau, is an issue worth exploring in future studies. Early evidence suggests that the meningeal lymphatic system may be implicated in the clearance of such elements [15, 184–186–187].

According to field experts, lymphosclerosis can be classified into four groups based on severity*:* (a) thin (translucent) and expandable lymphatic-vessel walls, and identifiable lumen (s0); (b) thin (white) and expandable lymphatic-vessel walls wall, and identifiable lumen (s1); (c) thick (white) nonexpandable lymphatic-vessel walls, and identifiable lumen (s2); and (d) very thick (white), nonexpandable lymphatic-vessel walls with nonidentifiable lumen (s3) [188].

Detailed anatomical and mechanistic studies are needed to further support our proposed model of lymphosclerosis (Fig. 1), and to determine which of the above four categories it fits. However, partial support comes from (a) recent evidence showing that amyloid-beta causes constriction of brain capillaries in a pericyte-mediated manner [26], and (b) from the close association of blood capillaries with lymphatic vessels, at least in the peripheral lymphatic system [166]. Additional support comes from studies on the basal meningeal lymphatic cells in ageing rodents, as outlined above [131] (based on findings from [189, 190]). Others have reported differences between dilated lymphatic vessels (ectatic type) and nondilated lymphatic vessels with thickened walls (contraction/sclerotic type). Therefore, two different characteristics of lymphatic vessels—diameter and sclerosis—should be evaluated [181]. Thus, in parallel to the potential presence of meningeal lymphatic hyperplasia in APOE4-mediated AD, additional evidence should be provided on the presence or lack of markers of meningeal lymphosclerosis.

To this end, it would be tempting to derive speculations from the observation that, in peripheral lymphedema, an arrest of lymphatic contraction occurs in late stages of the disease; notably, this arrest is characterized by a gradual decrease in the contraction amplitude (but unaltered frequency of contraction) of the collecting lymphatic vessels [190]. Nevertheless, the negative effects of the ligation of deep cervical lymph nodes, or of lymphosclerosis (if it is perceived as the functional analog of the mechanical ligation of lymph nodes) on AD may be disease-specific [186]. Notably, in the experimental autoimmune encephalitis model of MS, in which meningeal lymphatic vessels are not submitted to expansion (lymphangiogenesis), the obstruction of these vessels is linked to a reduction in brain reactive T-cell-mediated inflammation and amelioration of the disease phenotype [191, 192].

### Potential alternative explanations for our proposed conceptual framework

Another possibility could be that the altered neuroinflammatory response [21, 24, 193] and proinflammatory mediator levels (such as chitinase-3-like protein 1, and several cytokines, such as TNF-alpha, interleukin-1 receptor antagonist, and complement component C1q and 3) in the CSF of patients with AD [195–196] may be caused by abnormal microglial function [197]. Interestingly, although certain microglial types confer resistance to neurodegeneration [198], and higher resistance to certain regions, such as cerebellum and white matter [199], the so-called morphologically activated microglia exerts an influence on neurodegeneration that could be similar to the one caused by APOE4 [200]. Therefore, in line with recent findings on APOE4’s role in microglial activation [201], APOE4-mediated neuroinflammatory response and proinflammatory cytokines may ultimately influence CNS lymphatic-vessel drainage capacity, and disturb the composition and exchange of both CSF and ISF macromolecules, as well as CNS immunity.

### Framing of our conceptual framework into the broader evidence regarding AD pathogenesis

Given the lack of studies exploring the role of APOE4 in meningeal lymphatic function, additional evidence will be needed to explore the concepts presented here. Of note, our informed hypothesis on the APOE4-mediated meningeal lymphedema may not be entirely surprising, considering that (a) APOE4 may exercise its effects in a pathway that involves the vascular endothelial growth factor (VEGF), and VEGF upregulation can reverse APOE4 pathology [202], and (b) VEGF-C administration can restore meningeal lymphatic-vessel pathology in aged mice [15]. Given that *APOE4* is a principal genetic factor for AD, elucidating its role in AD remains pivotal, similarly to the roles of chief risk factors in other diseases (e.g., mechanistic investigations on the FTO region, which has the strongest genetic links to obesity [203]).

More broadly, our conceptual framework also raises the questions: why APOE4 has not been associated with peripheral edema? Why has APOE4 been discussed almost exclusively in relation to meningeal lymphedema? Based on data (not shown) that we extracted from the Human Protein Atlas [204], APOE seems to be predominantly expressed at high levels in cerebral cortex, hippocampus, caudate nucleus, and adrenal medulla. All these tissues share common embryological origins. Therefore, the downstream effects of APOE would most likely be pertinent to these tissues.

As previously reviewed [60], it is also still unclear how meningeal lymphatic vessels respond to, and also control, the high levels of amyloid-beta contained in the brain fluids of patients with AD. Possibilities include poor drainage of the CSF, decreased paravascular clearance through the glymphatic system, and/or decreased clearance through the meningeal lymphatic system [60]. These questions become now highly relevant in light of the fact that disruption of meningeal lymphatic vessels in an AD mouse model leads to amyloid-beta deposits in the meninges [15]. Thus, we suggest that future studies should clarify the involvement and mechanisms through which APOE4 influences amyloid-beta deposition in the meninges.

Unfortunately, no study in humans has yet linked levels of the well-established and emerging serum/CSF biomarkers of AD (i.e., tau protein, phosphorylated tau-181, neurogranin, chitinase-3-like protein 1, neurofilament light, synaptosomal-associated protein-25, amyloid-beta-40 and amyloid-beta-42 isoforms, visinin-like protein 1, and blood alpha-2 macroglobulin [193, 206–210–211]) with alterations of the meningeal lymphatic system. This could be clarified by using advanced microscopy techniques (e.g., cryo-electron microscopy) of *APOE4*-expressing cells to elucidate abnormalities in the morphology of blood vasculature and lymphatic systems. In addition, 7-tesla MRI imaging [129] could enable a comparison of vessel diameter between patients with AD with or without the *APOE4* isoform [129].

Lastly, given the single-nucleotide variation difference between *APOE3* and *APOE4* (Fig. 1), genome editing by CRISPR/Cas9, similar to applications in other neurological diseases (reviewed in [62, 212]), could offer novel opportunities for the study of APOE4-mediated pathogenetic mechanisms [44].

## Major challenges and limitations

The data and conceptual framework discussed here must be interpreted in light of the following considerations. First, regulation of the transcriptome in one cell type does not necessarily reflect the modulation of the same genes in neighboring cells. Therefore, caution should be applied on how change in the analyzed cells reflects those in actual meningeal lymphatic endothelial cells. Potentially, the APOE4-mediated effects on meningeal lymphatic cells could be the outcome of a crosstalk between intra- and intercellular interactions between lymphatic endothelial cells, as also observed in developmental stages of the fetal and postnatal period [213]. Second, the technical difficulties associated with the assessment of the differences in postmortem brain specimens between APOE4 vs. non-APOE4 patients with AD pose a major challenge in the proposed conceptual framework; for instance, histological fixation may affect the diameter of lymphatic vessels, suggesting that rapid freezing of tissues may be critical in future experiments. Third, the in vitro studies analyzed here compared the *APOE3-* with the *APOE4*-expressing cells; however, the feasible addition of a knock-in cell (e.g., with *APOE2* genotype) would have been a valuable control. Doing so would be essential to draw further conclusions concerning the differential effects of *APOE3* and *APOE4* on the expression of the analyzed genes.

An important clinical consideration is that some of the lymphatic-vessel-associated genes examined here may not be specific to the meningeal lymphatic vessels, and that large changes in their expression levels in lymphatic endothelial cells would cause lymphedema. However, there are no available data on the higher incidence of peripheral lymphedema in patients with mutated *APOE4*, or in patients with AD. The tissue-specific expression of *APOE* implies that the effects of APOE will most likely be exerted in brain-related tissues. Moreover, it would be crucial to link experimental data derived from this conceptual framework with impaired functional connectivity in AD, and with cellular surrogates of AD-related cognitive decline.

Given that peripheral lymphatic vessels in the small intestine contribute to the transport of lipids to the circulatory system [214], it is likely that meningeal lymphatic vessels similarly remove fatty acids from brain parenchyma. Although the mechanism for the metabolism of fatty acids in hyperactive neurons has been recently elucidated (according to this mechanism, fatty acids coupled with ApoE-positive lipid droplets are discarded from neurons, endocytosed by neighboring astrocytes, and metabolized through oxidative phosphorylation, ultimately leading to activation of molecular pathways to overcome fatty acid toxicity, and, in turn, protect neuronal function [215]), its relevance for AD neurodegeneration requires further investigation. The above is significant, granted that the majority of differences in the brain transcriptome of patients with AD can be attributed to alterations of gene expression in excitatory neurons (along with those in oligodendrocytes) [216]. However, whether the potential role of meningeal lymphatic vessels in brain fatty acid metabolism is affected by APOE4 (both with regard to APOE4 lipid droplets and our concept on meningeal lymphatic-vessel architecture in APOE4-related AD) might be hard to interpret.

We also wish to point out that conducting single-cell studies from the prefrontal cortex of patients with AD (as in [216]) may not be directly relevant to meningeal lymphatic-vessel physiology, given that meningeal lymphatic cells do not cross the brain parenchyma but, most likely, receive fluid from the glymphatic system that does communicate with the brain parenchyma (for a review on CNS vasculature, see [217]). This lack of relevance could be attributed to (a) the fact that only brain parenchyma-traversing vascular structures exist in the prefrontal cortex, (b) technical challenges, because of the small, statistically underpowered number of each category of cells, and (c) the difficulty to control for common factors, such as age and gender. Of note, we conducted a preliminary reanalysis on the above single-cell transcriptomics study [216], where we mapped the *APOE* genotypes to individual cells. This reanalysis revealed a bias of *APOE4* allele-carrying patients for higher Braak stages, a method to classify the severity of AD based on autopsy findings. Notably, no samples with Braak stage 1 or 2 have the *APOE4* allele, and no non-*APOE4* samples have Braak stage VI. Mitigation of this bias might be possible in more abundant cell types (e.g., microglia, neurons, astrocytes, and oligodendrocytes) by limiting the analysis to the middle of severity level (i.e., where both genotypes are present). However, the latter approach is not equally possible in the two major cell populations of interest, namely pericytes and endothelial cells, because of their much smaller numbers (Supplementary File 3). Collectively, the above single-cell transcriptomic study [216] might have been biased, given that a) all APOE4 samples were also high-severity samples (as assessed using Braak stages), and b) only AD samples were examined, with no healthy controls but mere separation based on no or little pathology group and mild-to-severe pathology group.

Moreover, with regards to recent findings on basal meningeal lymphatic vessels [131], distinguishing the potential effects of APOE isoforms on basal vs. dorsal meningeal lymphatic vessels would also be of importance. Finally, in light of the very recently deciphered molecular anatomical connections between meninges and the skull, deciphering the complexity of meningeal architecture and how the latter could affect processes of neuroinflammation and neurodegeneration (including AD) will be of crucial importance [218].

## Conclusions and future perspectives

The study of meningeal lymphatics will hopefully improve our understanding of AD. Indeed, the evidence discussed here raises fascinating questions about the connection between APOE4 and the meningeal lymphatic system in AD. Deciphering the role of APOE4, the strongest known genetic link to AD, in the meningeal lymphatic system, could reveal a missing link in our understanding of the etiology and pathology of AD. The suggested association between APOE4 and molecules of the VEGF pathway, if further validated, could provide further insights into the demonstrated link between VEGF ligand and receptor genes with the cognitive decline and neuropathology of AD, although the precise mechanisms involved remain to be investigated [219].

More broadly, by considering (a) the recent observations on capillary vasoconstriction in AD [26], (b) the neuronal/astrocytic metabolism of fatty acids coupled with the established role of lymphatic vessels (at least in the peripheral system) for fatty acid transportation [215], (c) the inverse epidemiologic associations of AD with diets with low input of saturated fats (e.g., Mediterranean diet) [220], and (d) the absence of AD-like alterations in nonhuman primates [221], we could conceptually describe late-onset AD as an evolutionarily human-specific disease, in which several epidemiologic factors (such as modern Western lifestyle) exert a profound impact on a human-specific variant *APOE4*. In addition, lymphatic vessels share common features with other types of vessels; therefore, this conceptual framework on meningeal lymphatic cells (especially its aspect on lymphosclerosis) could be aligned with previous calls for further studies addressing how environmental factors affect, in both common and distinct ways, arterial (atherosclerosis), and venous capillaries (capillary vasoconstriction) in the skull region [26, 222].

Our conceptual framework, if further verified by additional, intensive mechanistic studies, could serve as a prelude to the development of CSF-implicating therapeutics in AD and other neurodegenerative disorders. Indeed, recent studies have demonstrated a broad, previously unexpected role of CSF in various brain pathologies [223], while intrathecal administration of medications was employed successfully in major neurogenetic disorders [224]. Therefore, we speculate that intrathecal administration of VEGF or other agents might restore normal anatomy and function of meningeal lymphatic vessels in AD, allowing passage of glymphatic and lymphatic fluid into the cervical lymph nodes and proper processing of its content by the resident FDCs. Interestingly, successful therapies involving cervical and other lymph nodes, potentially including FDCs, have been applied in other neurological diseases (e.g., the use of fingolimod in MS [225]).

We hope that this novel conceptual framework, coupled with previous and future findings, may help develop the notion of meningeal lymphedema and lymphosclerosis in APOE4-related AD. In a broader context, informed hypotheses such as this could assist in the integration of epidemiologic data (such as the role of low-lipid diets in AD) with molecular signaling data (APOE4-donwstream events). Finally, our approach highlights the power of reanalyzing open data to produce new perspectives in the precision medicine era [167, 226].

### Supplementary information

Supplementary File 1

Supplementary File 2

Supplementary File 3

Figure S1

Figure S2

Figure S3

Figure S4

Figure S5

Supplementary Figures and Tables Legends

## Appendix

### Identification of studies, and differential expression (re)analyses

To identify studies in which we could reanalyze samples with AD based on *APOE4* vs. *APOE3* phenotype, we searched for all studies present on the GEO (https://www.ncbi.nlm.nih.gov/geo/) until June 1, 2019 focusing on *APOE* in humans. More specifically, the URL to the query used to perform the search is https://www.ncbi.nlm.nih.gov/gds/?term=(apoe)%20AND%20%22Homo%20sapiens%22[porgn]%20AND%20(%22gse%22[Filter]). Overall, 22 datasets were identified (see Supplementary File 1 for included and excluded studies).

In general, a systematic meta-analysis of multiple studies pulled into a single dataset can increase the power to detect DE genes due to a larger sample size. However, the studies identified from the above GEO search originated from critically different experimental systems ranging from biopsies and iPSC lines to brain organoids, making these studies not comparable. Hence, forcing such different studies into a meta-analysis might not be meaningful. Indeed, combining datasets merely aiming to create a bigger dataset could lead to a number of biases [227]; as such, we considered all individual studies. For the description of specific studies identified through the query and exclusion criteria, see Supplementary File 1. For each of the respective studies, expression values were downloaded from the GEO repository. Whenever possible, using the preprocessed values deposited by the authors was preferred in favor of the raw data.

With regards to microarray-based studies, distribution of expression values across all genes within each sample was visually examined to establish whether the data had been normalized prior to submission. If necessary, raw intensities were log2-transformed and normalized using the quantile normalization method from the affyPLM package [228]. Probes without gene annotation were removed. Differential expression analysis was conducted using limma package [228]. Gene expression was modeled jointly for all samples in a given study, for which the *APOE* genotype status was available. The test contrast was set between samples containing at least one *APOE4* allele (*e4* group) and those containing no such allele (*no e4* group). Additional variables, such as Braak stage, gender, and age, were included in the model, whenever reported. To take into account the false discovery rate, adjusted *p* value was computed by Benjamini–Hochberg method on the genes of interest present within the study [229].

With regards to RNA-Seq studies, gene count matrices were downloaded from the GEO repository (based on their accession numbers from corresponding studies), and processed using edgeR package [230]. Genes with low expression levels were removed, as a result of our requirement for, at least five reads, in at least three samples. In the study GSE125050, which contained several samples with low library sizes, we additionally removed samples that had less than 10,000 genes with at least five reads, in order to ensure a minimal library complexity. Normalization factors were then calculated using the trimmed mean of *M* values method in edgeR package [230]. These normalization factors were used to calculate effective library sizes, in turn used to calculate normalized CPM values for genes of interest and visualized as boxplots and a heatmap. Differential expression testing was carried out using edgeR’s quasi-likelihood framework [231]. Setting of test contrasts and inclusion of additional variables were conducted, as described above for microarray-based studies. Raw *p* values were adjusted for multiple testing by applying the Benjamini–Hochberg method [229] on the genes of interest.

Concerning the analysis of SAGE-based dataset (GSE6677), mappings between SAGE tags and genes were downloaded from SAGEmap database (ftp://ftp.ncbi.nlm.nih.gov/pub/sage/mappings/SAGEmap_Hs_NlaIII_10_best.gz). No statistical analysis of the data was performed due to the lack of replication. Expression was visualized using scatter plots. For genes that had more than one associated tag, all counts for tags were plotted, and color was used to distinguish between different tags within the same gene.

With regard to the single-cell RNA-Seq studies, single-nucleus RNA-Seq profiling data from prefrontal cortex of patients with AD were obtained from the ROSMAP study (https://www.synapse.org/#!Synapse:syn18485175), after approval of request from the synapse access and compliance team, and in alignment with a previous study [232]. Clinical information for the participants, including the *APOE* genotype, was obtained from https://www.synapse.org/#!Synapse:syn3191087. We assigned the *APOE* genotype to each cell by matching via patient ID and calculated number of cells with a given genotype within each cell type.

## References

[1] World Health Organization. WHO guidelines approved by the Guidelines Review Committee. Risk reduction of cognitive decline and dementia: WHO Guidelines. Geneva: World Health Organization; 2019. 31219687

[2] Masters CL, Bateman R, Blennow K, Rowe CC, Sperling RA, Cummings JL (2015). Alzheimer’s disease. Nat Rev Dis Prim.

[3] Jack CR, Therneau TM, Weigand SD, Wiste HJ, Knopman DS, Vemuri P (2019). Prevalence of biologically vs clinically defined Alzheimer spectrum entities using the National Institute on Aging–Alzheimer’s Association research framework. JAMA Neurol.

[4] Devi G (2018). Alzheimer’s disease in physicians—assessing professional competence and tempering stigma. N Engl J Med.

[5] Scheltens NM, Galindo-Garre F, Pijnenburg YA, van der Vlies AE, Smits LL, Koene T (2016). The identification of cognitive subtypes in Alzheimer’s disease dementia using latent class analysis. J Neurol Neurosurg Psychiatry.

[6] Thomas KR, Bangen KJ, Weigand AJ, Edmonds EC, Wong CG, Cooper S, et al. Objective subtle cognitive difficulties predict future amyloid accumulation and neurodegeneration. Neurology. 2020 Jan 28;94:e397–e406. 10.1212/WNL.0000000000008838 PMC7079691 31888974

[7] Bellou V, Belbasis L, Tzoulaki I, Middleton LT, Ioannidis JPA, Evangelou E (2017). Systematic evaluation of the associations between environmental risk factors and dementia: an umbrella review of systematic reviews and meta-analyses. Alzheimer’s Dement.

[8] Gassen NC, Chrousos GP, Binder EB, Zannas AS (2017). Life stress, glucocorticoid signaling, and the aging epigenome: implications for aging-related diseases. Neurosci Biobehav Rev.

[9] Nicolaides NC, Kyratzi E, Lamprokostopoulou A, Chrousos GP, Charmandari E (2015). Stress, the stress system and the role of glucocorticoids. Neuroimmunomodulation.

[10] Canet G, Hernandez C, Zussy C, Chevallier N, Desrumaux C, Givalois L (2019). Is AD a stress-related disorder? Focus on the HPA axis and its promising therapeutic targets. Front Aging Neurosci.

[11] Bangasser D, Dong H, Carroll J, Plona Z, Ding H, Rodriguez L (2017). Corticotropin-releasing factor overexpression gives rise to sex differences in Alzheimer’s disease-related signaling. Mol Psychiatry.

[12] Fischer R, Maier O (2015). Interrelation of oxidative stress and inflammation in neurodegenerative disease: role of TNF. Oxid Med Cell Longev.

[13] Lecca D, Bader M, Tweedie D, Hoffman AF, Jung YJ, Hsueh SC, et al. (-)-Phenserine and the prevention of pre-programmed cell death and neuroinflammation in mild traumatic brain injury and Alzheimer’s disease challenged mice. Neurobio Dis. 2019;130:104528. 10.1016/j.nbd.2019.104528 PMC6716152 31295555

[14] Ransohoff RM (2016). How neuroinflammation contributes to neurodegeneration. Science.

[15] Da Mesquita S, Louveau A, Vaccari A, Smirnov I, Cornelison RC, Kingsmore KM (2018). Functional aspects of meningeal lymphatics in ageing and Alzheimer’s disease. Nature.

[16] Michelson D, Stone L, Galliven E, Magiakou MA, Chrousos GP, Sternberg EM (1994). Multiple sclerosis is associated with alterations in hypothalamic-pituitary-adrenal axis function. J Clin Endocrinol Metab.

[17] Pervanidou P, Chrousos GP. Neuroendocrinology of post-traumatic stress disorder. In: Progress in brain research, Vol. 182. Elsevier; Amsterdam, Netherlands; 2010. p. 149–60. 10.1016/S0079-6123(10)82005-9 20541663

[18] Le CP, Nowell CJ, Kim-Fuchs C, Botteri E, Hiller JG, Ismail H (2016). Chronic stress in mice remodels lymph vasculature to promote tumour cell dissemination. Nat Commun.

[19] Chrousos GP (1995). The hypothalamic-pituitary-adrenal axis and immune-mediated inflammation. N Engl J Med.

[20] Chrousos GP (2009). Stress and disorders of the stress system. Nat Rev Endocrinol.

[21] Gjoneska E, Pfenning AR, Mathys H, Quon G, Kundaje A, Tsai LH (2015). Conserved epigenomic signals in mice and humans reveal immune basis of Alzheimer’s disease. Nature.

[22] Heckmann BL, Teubner BJW, Tummers B, Boada-Romero E, Harris L, Yang M, et al. LC3-associated endocytosis facilitates beta-amyloid clearance and mitigates neurodegeneration in murine Alzheimer’s disease. Cell. 2019;178:536–51.e14. 10.1016/j.cell.2019.05.056 PMC6689199 31257024

[23] Heneka MT, Kummer MP, Stutz A, Delekate A, Schwartz S, Vieira-Saecker A (2013). NLRP3 is activated in Alzheimer’s disease and contributes to pathology in APP/PS1 mice. Nature.

[24] Hong S, Beja-Glasser VF, Nfonoyim BM, Frouin A, Li S, Ramakrishnan S (2016). Complement and microglia mediate early synapse loss in Alzheimer mouse models. Science.

[25] Liu C-C, Kanekiyo T, Xu H, Bu G (2013). Apolipoprotein E and Alzheimer disease: risk, mechanisms and therapy. Nat Rev Neurol.

[26] Nortley R, Korte N, Izquierdo P, Hirunpattarasilp C, Mishra A, Jaunmuktane Z, et al. Amyloid beta oligomers constrict human capillaries in Alzheimer’s disease via signaling to pericytes. Science. 2019;365:eaav9518. 10.1126/science.aav9518. PMC6658218 31221773

[27] Mahley RW (2017). Apolipoprotein E: remarkable protein sheds light on cardiovascular and neurological diseases. Clin Chem.

[28] Hudry E, Klickstein J, Cannavo C, Jackson R, Muzikansky A, Gandhi S, et al. Opposing roles of apolipoprotein E in aging and neurodegeneration. Life Sci Alliance. 2019;2:e201900325. 10.26508/lsa.201900325 PMC6374993 30760557

[29] Arboleda-Velasquez JF, Lopera F, O’Hare M, Delgado-Tirado S, Marino C, Chmielewska N, et al. Resistance to autosomal dominant Alzheimer’s disease in an APOE3 Christchurch homozygote: a case report. Nat Med. 2019;25:1680–83. 10.1038/s41591-019-0611-3 PMC6898984 31686034

[30] Weissberger GH, Nation DA, Nguyen CP, Bondi MW, Han SD (2018). Meta-analysis of cognitive ability differences by apolipoprotein e genotype in young humans. Neurosci Biobehav Rev.

[31] Ritchie SJ, Hill WD, Marioni RE, Davies G, Hagenaars SP, Harris SE, et al. Polygenic predictors of age-related decline in cognitive ability. Mol Psychiatry. 2019. 10.1038/s41380-019-0372-x. [Online ahead of print]. 10.1038/s41380-019-0372-x PMC7515838 30760887

[32] Glorioso CA, Pfenning AR, Lee SS, Bennett DA, Sibille EL, Kellis M, et al. Rate of brain aging and APOE epsilon4 are synergistic risk factors for Alzheimer’s disease. Life Sci Alliance. 2019;2:e201900303. 10.26508/lsa.201900303 PMC6537750 31133613

[33] Xian X, Pohlkamp T, Durakoglugil MS, Wong CH, Beck JK, Lane-Donovan C, et al. Reversal of ApoE4-induced recycling block as a novel prevention approach for Alzheimer’s disease. eLife. 2018;7:e40048. 10.7554/eLife.40048 PMC6261251 30375977

[34] Ritchie K, Carrière I, Ritchie C, Berr C, Artero S, Ancelin M-L (2010). Designing prevention programmes to reduce incidence of dementia: prospective cohort study of modifiable risk factors. BMJ.

[35] De Luca V, Spalletta G, Souza RP, Graff A, Bastos-Rodrigues L, Camargos Bicalho MA (2019). Definition of late onset Alzheimer’s disease and anticipation effect of genome-wide significant risk variants: pilot study of the APOE e4 allele. Neuropsychobiology.

[36] Liao F, Li A, Xiong M, Bien-Ly N, Jiang H, Zhang Y (2018). Targeting of nonlipidated, aggregated apoE with antibodies inhibits amyloid accumulation. J Clin Investig.

[37] Tulloch J, Leong L, Chen S, Keene CD, Millard SP, Shutes-David A (2018). APOE DNA methylation is altered in Lewy body dementia. Alzheimer’s Dement.

[38] Geiger JT, Ding J, Crain B, Pletnikova O, Letson C, Dawson TM (2016). Next-generation sequencing reveals substantial genetic contribution to dementia with Lewy bodies. Neurobiol Dis.

[39] Dickson DW, Heckman MG, Murray ME, Soto AI, Walton RL, Diehl NN (2018). APOE epsilon4 is associated with severity of Lewy body pathology independent of Alzheimer pathology. Neurology.

[40] Tsuang D, Leverenz JB, Lopez OL, Hamilton RL, Bennett DA, Schneider JA (2013). APOE epsilon4 increases risk for dementia in pure synucleinopathies. JAMA Neurol.

[41] Lin YF, Smith AV, Aspelund T, Betensky RA, Smoller JW, Gudnason V (2019). Genetic overlap between vascular pathologies and Alzheimer’s dementia and potential causal mechanisms. Alzheimer’s Dement.

[42] Skillback T, Lautner R, Mattsson N, Schott JM, Skoog I, Nagga K (2018). Apolipoprotein E genotypes and longevity across dementia disorders. Alzheimer’s Dement.

[43] Gandy S, Dekosky ST (2012). APOE epsilon4 status and traumatic brain injury on the gridiron or the battlefield. Sci Transl Med.

[44] Safieh M, Korczyn AD, Michaelson DM (2019). ApoE4: an emerging therapeutic target for Alzheimer’s disease. BMC Med.

[45] Lamar M, Yu L, Rubin LH, James BD, Barnes LL, Farfel JM (2019). APOE genotypes as a risk factor for age-dependent accumulation of cerebrovascular disease in older adults. Alzheimer’s Dement.

[46] Wennberg AM, Tosakulwong N, Lesnick TG, Murray ME, Whitwell JL, Liesinger AM (2018). Association of apolipoprotein E epsilon4 with transactive response DNA-binding protein 43. JAMA Neurol.

[47] Velez JI, Lopera F, Sepulveda-Falla D, Patel HR, Johar AS, Chuah A (2016). APOE*E2 allele delays age of onset in PSEN1 E280A Alzheimer’s disease. Mol Psychiatry.

[48] Hauser PS, Ryan RO (2013). Impact of apolipoprotein E on Alzheimer’s disease. Curr Alzheimer Res.

[49] Moreno DJ, Pino S, Rios A, Lopera F, Ostos H, Via M (2017). Genetic Ancestry And Susceptibility To Late-onset Alzheimer disease (LOAD) in the admixed Colombian population. Alzheimer Dis Assoc Disord.

[50] Reitz C, Jun G, Naj A, Rajbhandary R, Vardarajan BN, Wang LS (2013). Variants in the ATP-binding cassette transporter (ABCA7), apolipoprotein E 4,and the risk of late-onset Alzheimer disease in African Americans. JAMA.

[51] Maestre G, Ottman R, Stern Y, Gurland B, Chun M, Tang MX (1995). Apolipoprotein E and Alzheimer’s disease: ethnic variation in genotypic risks. Ann Neurol.

[52] Mattsson N, Groot C, Jansen WJ, Landau SM, Villemagne VL, Engelborghs S (2018). Prevalence of the apolipoprotein E epsilon4 allele in amyloid beta positive subjects across the spectrum of Alzheimer’s disease. Alzheimer’s Demen.

[53] Duara R, Loewenstein DA, Lizarraga G, Adjouadi M, Barker WW, Greig-Custo MT (2019). Effect of age, ethnicity, sex, cognitive status and APOE genotype on amyloid load and the threshold for amyloid positivity. NeuroImage Clin.

[54] Yamazaki Y, Zhao N, Caulfield TR, Liu CC, Bu G. Apolipoprotein E and Alzheimer disease: pathobiology and targeting strategies. Nat Rev Neurol. 2019;15:501–18. 10.1038/s41582-019-0228-7 PMC7055192 31367008

[55] Tachibana M, Holm ML, Liu CC, Shinohara M, Aikawa T, Oue H (2019). APOE4-mediated amyloid-beta pathology depends on its neuronal receptor LRP1. J Clin Investig.

[56] Yan Q, Nho K, Del-Aguila JL, Wang X, Risacher SL, Fan KH, et al. Genome-wide association study of brain amyloid deposition as measured by Pittsburgh Compound-B (PiB)-PET imaging. Mol Psychiatry. 2018. 10.1038/s41380-018-0246-7. [Online ahead of print]. 10.1038/s41380-018-0246-7 PMC6219464 30361487

[57] Lim YY, Villemagne VL, Laws SM, Pietrzak RH, Snyder PJ, Ames D (2015). APOE and BDNF polymorphisms moderate amyloid beta-related cognitive decline in preclinical Alzheimer’s disease. Mol Psychiatry.

[58] Kara E, Marks JD, Roe AD, Commins C, Fan Z, Calvo-Rodriguez M (2018). A flow cytometry-based in vitro assay reveals that formation of apolipoprotein E (ApoE)-amyloid beta complexes depends on ApoE isoform and cell type. J Biol Chem.

[59] Hori Y, Hashimoto T, Nomoto H, Hyman BT, Iwatsubo T (2015). Role of apolipoprotein E in beta-amyloidogenesis: isoform-specific effects on protofibril to fibril conversion of abeta in vitro and brain abeta deposition in vivo. J Biol Chem.

[60] Louveau A, Da Mesquita S, Kipnis J (2016). Lymphatics in neurological disorders: a neuro-lympho-vascular component of multiple sclerosis and Alzheimer’s disease?. Neuron.

[61] Liu CC, Zhao N, Fu Y, Wang N, Linares C, Tsai CW (2017). ApoE4 accelerates early seeding of amyloid pathology. Neuron.

[62] Mentis AF (2016). Epigenomic engineering for Down syndrome. Neurosci Biobehav Rev.

[63] Wiseman FK, Pulford LJ, Barkus C, Liao F, Portelius E, Webb R (2018). Trisomy of human chromosome 21 enhances amyloid-β deposition independently of an extra copy of APP. Brain.

[64] Hithersay R, Startin CM, Hamburg S, Mok KY, Hardy J, Fisher EMC, et al. Association of dementia with mortality among adults with Down syndrome older than 35 years. JAMA Neurol. 2019;76:152–60. 10.1001/jamaneurol.2018.3616 PMC6439956 30452522

[65] Tapia-Rojas C, Cabezas-Opazo F, Deaton CA, Vergara EH, Johnson GVW, Quintanilla RA (2019). It’s all about tau. Prog Neurobiol.

[66] Shi Y, Yamada K, Liddelow SA, Smith ST, Zhao L, Luo W (2017). ApoE4 markedly exacerbates tau-mediated neurodegeneration in a mouse model of tauopathy. Nature.

[67] Wadhwani AR, Affaneh A, Van Gulden S, Kessler JA. Neuronal apolipoprotein E4 increases cell death and p-tau release in Alzheimer’s disease. Ann Neurol. 2019;85:726–39. 10.1002/ana.25455 PMC8123085 30840313

[68] Peng KY, Perez-Gonzalez R, Alldred MJ, Goulbourne CN, Morales-Corraliza J, Saito M (2019). Apolipoprotein E4 genotype compromises brain exosome production. Brain.

[69] Ding Y, Huang L, Xian X, Yuhanna IS, Wasser CR, Frotscher M (2016). Loss of reelin protects against atherosclerosis by reducing leukocyte-endothelial cell adhesion and lesion macrophage accumulation. Sci Signal.

[70] Lane-Donovan C, Philips GT, Wasser CR, Durakoglugil MS, Masiulis I, Upadhaya A (2015). Reelin protects against amyloid beta toxicity in vivo. Sci Signal.

[71] Setti SE, Hunsberger HC, Reed MN (2017). Alterations in hippocampal activity and Alzheimer’s disease. Transl Issues Psychol Sci.

[72] Šišková Z, Justus D, Kaneko H, Friedrichs D, Henneberg N, Beutel T (2014). Dendritic structural degeneration is functionally linked to cellular hyperexcitability in a mouse model of Alzheimer’s disease. Neuron.

[73] Najm R, Jones EA, Huang Y (2019). Apolipoprotein E4, inhibitory network dysfunction, and Alzheimer’s disease. Mol Neurodegener.

[74] Vitek MP, Brown CM, Colton CA (2009). APOE genotype-specific differences in the innate immune response. Neurobiol Aging.

[75] Heneka MT, Golenbock DT, Latz E (2015). Innate immunity in Alzheimer’s disease. Nat Immunol.

[76] Sala Frigerio C, Wolfs L, Fattorelli N, Thrupp N, Voytyuk I, Schmidt I (2019). The major risk factors for Alzheimer’s disease: age, sex, and genes modulate the microglia response to abeta plaques. Cell Rep..

[77] Dardiotis E, Siokas V, Pantazi E, Dardioti M, Rikos D, Xiromerisiou G (2017). A novel mutation in TREM2 gene causing Nasu-Hakola disease and review of the literature. Neurobiol Aging.

[78] Krasemann S, Madore C, Cialic R, Baufeld C, Calcagno N, El Fatimy R (2017). The TREM2-APOE pathway drives the transcriptional phenotype of dysfunctional microglia in neurodegenerative diseases. Immunity.

[79] Yeh FL, Wang Y, Tom I, Gonzalez LC, Sheng M (2016). TREM2 binds to apolipoproteins, including APOE and CLU/APOJ, and thereby facilitates uptake of amyloid-beta by microglia. Neuron.

[80] Parhizkar S, Arzberger T, Brendel M, Kleinberger G, Deussing M, Focke C (2019). Loss of TREM2 function increases amyloid seeding but reduces plaque-associated ApoE. Nat Neurosci.

[81] Sims R, Van Der Lee SJ, Naj AC, Bellenguez C, Badarinarayan N, Jakobsdottir J (2017). Rare coding variants in PLCG2, ABI3, and TREM2 implicate microglial-mediated innate immunity in Alzheimer’s disease. Nat Genet.

[82] Neumann H, Daly MJ (2013). Variant TREM2 as risk factor for Alzheimer’s disease. N Engl J Med.

[83] Benitez BA, Cruchaga C (2013). TREM2 and neurodegenerative disease. N Engl J Med.

[84] Guerreiro R, Wojtas A, Bras J, Carrasquillo M, Rogaeva E, Majounie E (2013). TREM2 variants in Alzheimer’s disease. N Engl J Med.

[85] Slattery CF, Beck JA, Harper L, Adamson G, Abdi Z, Uphill J (2014). R47H TREM2 variant increases risk of typical early-onset Alzheimer’s disease but not of prion or frontotemporal dementia. Alzheimer’s Dement.

[86] Jonsson T, Stefansson H, Steinberg S, Jonsdottir I, Jonsson PV, Snaedal J (2013). Variant of TREM2 associated with the risk of Alzheimer’s disease. N Engl J Med.

[87] Sweeney MD, Montagne A, Sagare AP, Nation DA, Schneider LS, Chui HC (2019). Vascular dysfunction-The disregarded partner of Alzheimer’s disease. Alzheimer’s Dement.

[88] Merlini M, Rafalski VA, Rios Coronado PE, Gill TM, Ellisman M, Muthukumar G (2019). Fibrinogen induces microglia-mediated spine elimination and cognitive impairment in an Alzheimer’s disease model. Neuron.

[89] Jack CR, Holtzman DM (2013). Biomarker modeling of Alzheimer’s disease. Neuron.

[90] Scheltens P, Blennow K, Breteler MM, de Strooper B, Frisoni GB, Salloway S (2016). Alzheimer’s disease. Lancet.

[91] Leuzy A, Chiotis K, Lemoine L, Gillberg PG, Almkvist O, Rodriguez-Vieitez E, et al. Tau PET imaging in neurodegenerative tauopathies-still a challenge. Mol Psychiatry. 2019;24:1112–34. 10.1038/s41380-018-0342-8 PMC6756230 30635637

[92] Kapogiannis D, Mustapic M, Shardell MD, Berkowitz ST, Diehl TC, Spangler RD, et al. Association of extracellular vesicle biomarkers with Alzheimer disease in the Baltimore Longitudinal Study of Aging. JAMA Neurol. 2019;76:1340–51. 10.1001/jamaneurol.2019.2462 PMC6632160 31305918

[93] Nation DA, Sweeney MD, Montagne A, Sagare AP, D’Orazio LM, Pachicano M (2019). Blood-brain barrier breakdown is an early biomarker of human cognitive dysfunction. Nat Med.

[94] Slot RER, Sikkes SAM, Berkhof J, Brodaty H, Buckley R, Cavedo E (2019). Subjective cognitive decline and rates of incident Alzheimer’s disease and non-Alzheimer’s disease dementia. Alzheimer’s Dement.

[95] Bell RD, Winkler EA, Singh I, Sagare AP, Deane R, Wu Z (2012). Apolipoprotein E controls cerebrovascular integrity via cyclophilin A. Nature.

[96] Halliday MR, Pomara N, Sagare AP, Mack WJ, Frangione B, Zlokovic BV (2013). Relationship between cyclophilin a levels and matrix metalloproteinase 9 activity in cerebrospinal fluid of cognitively normal apolipoprotein e4 carriers and blood-brain barrier breakdown. JAMA Neurol.

[97] Azarpazhooh MR, Avan A, Cipriano LE, Munoz DG, Sposato LA, Hachinski V (2018). Concomitant vascular and neurodegenerative pathologies double the risk of dementia. Alzheimer’s Dement.

[98] Kapasi A, DeCarli C, Schneider JA (2017). Impact of multiple pathologies on the threshold for clinically overt dementia. Acta Neuropathol.

[99] Rabin JS, Yang HS, Schultz AP, Hanseeuw BJ, Hedden T, Viswanathan A (2019). Vascular risk and beta-amyloid are synergistically associated with cortical tau. Ann Neurol.

[100] Szoeke C, Goodwill AM, Gorelik A, Dennerstein L, Caeyenberghs K, Simpson S, et al. Apolipoprotein E4 mediates the association between midlife dyslipidemia and cerebral amyloid in aging women. J Alzheimer’s Dis. 2019;68:105–14. 10.3233/JAD-180815 30689578

[101] Zhang Y, Song W (2017). Islet amyloid polypeptide: another key molecule in Alzheimer’s pathogenesis?. Prog Neurobiol.

[102] Robert J, Button EB, Yuen B, Gilmour M, Kang K, Bahrabadi A, et al. Clearance of beta-amyloid is facilitated by apolipoprotein E and circulating high-density lipoproteins in bioengineered human vessels. eLife. 2017;6:e29595. 10.7554/eLife.29595 PMC5634784 28994390

[103] Castellano JM, Deane R, Gottesdiener AJ, Verghese PB, Stewart FR, West T (2012). Low-density lipoprotein receptor overexpression enhances the rate of brain-to-blood Abeta clearance in a mouse model of beta-amyloidosis. Proc Natl Acad Sci USA.

[104] Ma Q, Zhao Z, Sagare AP, Wu Y, Wang M, Owens NC (2018). Blood-brain barrier-associated pericytes internalize and clear aggregated amyloid-beta42 by LRP1-dependent apolipoprotein E isoform-specific mechanism. Mol Neurodegener.

[105] Alitalo K, Tammela T, Petrova TV (2005). Lymphangiogenesis in development and human disease. Nature.

[106] Tammela T, Alitalo K (2010). Lymphangiogenesis: molecular mechanisms and future promise. Cell.

[107] Klotz L, Norman S, Vieira JM, Masters M, Rohling M, Dube KN (2015). Cardiac lymphatics are heterogeneous in origin and respond to injury. Nature.

[108] Pichol-Thievend C, Betterman KL, Liu X, Ma W, Skoczylas R, Lesieur E, et al. A blood capillary plexus-derived population of progenitor cells contributes to genesis of the dermal lymphatic vasculature during embryonic development. Development. 2018;145:dev160184. 10.1242/dev.160184 PMC6001371 29773646

[109] Martinez-Corral I, Ulvmar MH, Stanczuk L, Tatin F, Kizhatil K, John SW (2015). Nonvenous origin of dermal lymphatic vasculature. Circ Res.

[110] Lukic IK, Gluncic V, Ivkic G, Hubenstorf M, Marusic A (2003). Virtual dissection: a lesson from the 18th century. Lancet.

[111] Foldi M, Gellert A, Kozma M, Poberai M, Zoltan OT, Csanda E (1966). New contributions to the anatomical connections of the brain and the lymphatic system. Acta Anat.

[112] Prineas JW (1979). Multiple sclerosis: presence of lymphatic capillaries and lymphoid tissue in the brain and spinal cord. Science.

[113] Andres KH, von During M, Muszynski K, Schmidt RF (1987). Nerve fibres and their terminals of the dura mater encephali of the rat. Anat Embryol.

[114] Gausas RE, Daly T, Fogt F (2007). D2-40 expression demonstrates lymphatic vessel characteristics in the dural portion of the optic nerve sheath. Ophthalmic Plast Reconstr Surg.

[115] Furukawa M, Shimoda H, Kajiwara T, Kato S, Yanagisawa S (2008). Topographic study on nerve-associated lymphatic vessels in the murine craniofacial region by immunohistochemistry and electron microscopy. Biomed Res.

[116] Waggener JD, Beggs J (1967). The membranous coverings of neural tissues: an electron microscopy study. J Neuropathol Exp Neurol.

[117] Alders M, Al-Gazali L, Cordeiro I, Dallapiccola B, Garavelli L, Tuysuz B (2014). Hennekam syndrome can be caused by FAT4 mutations and be allelic to Van Maldergem syndrome. Hum Genet.

[118] Berton M, Lorette G, Baulieu F, Lagrue E, Blesson S, Cambazard F (2015). Generalized lymphedema associated with neurologic signs (GLANS) syndrome: a new entity?. J Am Acad Dermatol.

[119] Louveau A, Smirnov I, Keyes TJ, Eccles JD, Rouhani SJ, Peske JD (2015). Structural and functional features of central nervous system lymphatic vessels. Nature.

[120] Antila S, Karaman S, Nurmi H, Airavaara M, Voutilainen MH, Mathivet T (2017). Development and plasticity of meningeal lymphatic vessels. J Exp Med.

[121] Aspelund A, Antila S, Proulx ST, Karlsen TV, Karaman S, Detmar M (2015). A dural lymphatic vascular system that drains brain interstitial fluid and macromolecules. J Exp Med.

[122] Sun BL, Wang LH, Yang T, Sun JY, Mao LL, Yang MF (2018). Lymphatic drainage system of the brain: a novel target for intervention of neurological diseases. Prog Neurobiol.

[123] Frederick N, Louveau A (2020). Meningeal lymphatics, immunity and neuroinflammation. Curr Opin Neurobiol.

[124] Sandrone S, Moreno-Zambrano D, Kipnis J, van Gijn JA (2019). (delayed) history of the brain lymphatic system. Nat Med.

[125] Aspelund A, Tammela T, Antila S, Nurmi H, Leppanen VM, Zarkada G (2014). The Schlemm’s canal is a VEGF-C/VEGFR-3-responsive lymphatic-like vessel. J Clin Investig.

[126] Aselli G. De Lacteibus sive Lacteis Venis, Quarto Vasorum Mesarai corum Genere novo invento. Milan: Mediolani; 1627.

[127] Butler MG, Isogai S, Weinstein BM (2009). Lymphatic development. Birth defects Res Part C.

[128] Aspelund A, Robciuc MR, Karaman S, Makinen T, Alitalo K (2016). Lymphatic system in cardiovascular medicine. Circ Res.

[129] Absinta M, Ha SK, Nair G, Sati P, Luciano NJ, Palisoc M, et al. Human and nonhuman primate meninges harbor lymphatic vessels that can be visualized noninvasively by MRI. eLife. 2017;6:e29738. 10.7554/eLife.29738 PMC5626482 28971799

[130] Da Mesquita S, Fu Z, Kipnis J (2018). The meningeal lymphatic system: a new player in neurophysiology. Neuron.

[131] Ahn JH, Cho H, Kim JH, Kim SH, Ham JS, Park I, et al. Meningeal lymphatic vessels at the skull base drain cerebrospinal fluid. Nature. 2019;572:62–6. 10.1038/s41586-019-1419-5 31341278

[132] Bower NI, Koltowska K, Pichol-Thievend C, Virshup I, Paterson S, Lagendijk AK (2017). Mural lymphatic endothelial cells regulate meningeal angiogenesis in the zebrafish. Nat Neurosci.

[133] Iliff JJ, Wang M, Liao Y, Plogg BA, Peng W, Gundersen GA (2012). A paravascular pathway facilitates CSF flow through the brain parenchyma and the clearance of interstitial solutes, including amyloid beta. Sci Transl Med.

[134] Ma Q, Ineichen BV, Detmar M, Proulx ST (2017). Outflow of cerebrospinal fluid is predominantly through lymphatic vessels and is reduced in aged mice. Nat Commun.

[135] Kress BT, Iliff JJ, Xia M, Wang M, Wei HS, Zeppenfeld D (2014). Impairment of paravascular clearance pathways in the aging brain. Ann Neurol.

[136] Stern JNH, Yaari G, Vander Heiden JA, Church G, Donahue WF, Hintzen RQ (2014). B cells populating the multiple sclerosis brain mature in the draining cervical lymph nodes. Sci Transl Med.

[137] Palanichamy A, Apeltsin L, Kuo TC, Sirota M, Wang S, Pitts SJ (2014). Immunoglobulin class-switched B cells form an active immune axis between CNS and periphery in multiple sclerosis. Sci Transl Med.

[138] Mentis A-FA, Dardiotis E, Grigoriadis N, Petinaki E, Hadjigeorgiou GM (2017). Viruses and multiple sclerosis: from mechanisms and pathways to translational research opportunities. Mol Neurobiol.

[139] Mentis AFA, Dardiotis E, Grigoriadis N, Petinaki E, Hadjigeorgiou GM (2017). Viruses and endogenous retroviruses in multiple sclerosis: from correlation to causation. Acta Neurol Scand.

[140] Krautler NJ, Kana V, Kranich J, Tian Y, Perera D, Lemm D (2012). Follicular dendritic cells emerge from ubiquitous perivascular precursors. Cell.

[141] Dave RS, Jain P, Byrareddy SN (2018). Follicular dendritic cells of lymph nodes as human immunodeficiency virus/simian immunodeficiency virus reservoirs and insights on cervical lymph node. Front Immunol.

[142] Heesters BA, Lindqvist M, Vagefi PA, Scully EP, Schildberg FA, Altfeld M (2015). Follicular dendritic cells retain infectious HIV in cycling endosomes. PLoS Pathog.

[143] Dave RS, Sharma RK, Muir RR, Haddad E, Gumber S, Villinger F (2018). FDC: TFH interactions within cervical lymph nodes of SIV-infected rhesus macaques. J Neuroimmune Pharmacol.

[144] Xiang M, Grosso RA, Takeda A, Pan J, Bekkhus T, Brulois K, et al. A single-cell transcriptional roadmap of the mouse and human lymph node lymphatic vasculature. 2020. 10.1101/2019.12.31.892166. 10.3389/fcvm.2020.00052 PMC7204639 32426372

[145] Baloyannis SJ, Baloyannis IS (2012). The vascular factor in Alzheimer’s disease: a study in Golgi technique and electron microscopy. J Neurol Sci.

[146] Kimura T, Hashimura T, Miyakawa T (1991). Observations of microvessels in the brain with Alzheimer’s disease by the scanning electron microscopy. Jpn J Psychiatry Neurol.

[147] Sica D (2003). Calcium channel blocker-related periperal edema: can it be resolved?. J Clin Hypertens.

[148] de Jong DLK, de Heus RAA, Rijpma A, Donders R, Olde Rikkert MGM, Gunther M (2019). Effects of nilvadipine on cerebral blood flow in patients with Alzheimer disease. Hypertension.

[149] Lim HY, Rutkowski JM, Helft J, Reddy ST, Swartz MA, Randolph GJ (2009). Hypercholesterolemic mice exhibit lymphatic vessel dysfunction and degeneration. Am J Pathol.

[150] Broce IJ, Tan CH, Fan CC, Jansen I, Savage JE, Witoelar A, et al. Dissecting the genetic relationship between cardiovascular risk factors and Alzheimer’s disease. Acta Neuropathol. 2019;137:209–26. 10.1007/s00401-018-1928-6 PMC6358498 30413934

[151] Chen CY, Bertozzi C, Zou Z, Yuan L, Lee JS, Lu M (2012). Blood flow reprograms lymphatic vessels to blood vessels. J Clin Investig.

[152] Achariyar TM, Li B, Peng W, Verghese PB, Shi Y, McConnell E (2016). Glymphatic distribution of CSF-derived apoE into brain is isoform specific and suppressed during sleep deprivation. Mol Neurodegener.

[153] Oakley H, Cole SL, Logan S, Maus E, Shao P, Craft J (2006). Intraneuronal beta-amyloid aggregates, neurodegeneration, and neuron loss in transgenic mice with five familial Alzheimer’s disease mutations: potential factors in amyloid plaque formation. J Neurosci.

[154] Eide PK, Ringstad G (2015). MRI with intrathecal MRI gadolinium contrast medium administration: a possible method to assess glymphatic function in human brain. Acta Radiol Open.

[155] Louveau A, Plog BA, Antila S, Alitalo K, Nedergaard M, Kipnis J (2017). Understanding the functions and relationships of the glymphatic system and meningeal lymphatics. J Clin Investig.

[156] Fultz NE, Bonmassar G, Setsompop K, Stickgold RA, Rosen BR, Polimeni JR (2019). Coupled electrophysiological, hemodynamic, and cerebrospinal fluid oscillations in human sleep. Science.

[157] Xie L, Kang H, Xu Q, Chen MJ, Liao Y, Thiyagarajan M (2013). Sleep drives metabolite clearance from the adult brain. Science.

[158] Rasmussen MK, Mestre H, Nedergaard M (2018). The glymphatic pathway in neurological disorders. Lancet Neurol.

[159] Cao X, Xu H, Feng W, Su D, Xiao M (2018). Deletion of aquaporin-4 aggravates brain pathology after blocking of the meningeal lymphatic drainage. Brain Res Bull.

[160] Abbott NJ, Pizzo ME, Preston JE, Janigro D, Thorne RG (2018). The role of brain barriers in fluid movement in the CNS: is there a ‘glymphatic’ system?. Acta Neuropathol.

[161] Smith AJ, Yao X, Dix JA, Jin BJ, Verkman AS. Test of the ‘glymphatic’ hypothesis demonstrates diffusive and aquaporin-4-independent solute transport in rodent brain parenchyma. eLife. 2017;6:e27679. 10.7554/eLife.27679 PMC5578736 28826498

[162] Mestre H, Hablitz LM, Xavier AL, Feng W, Zou W, Pu T, et al. Aquaporin-4-dependent glymphatic solute transport in the rodent brain. eLife. 2018;7:e40070. 10.7554/eLife.40070 PMC6307855 30561329

[163] Lin YT, Seo J, Gao F, Feldman HM, Wen HL, Penney J (2018). APOE4 causes widespread molecular and cellular alterations associated with Alzheimer’s disease phenotypes in human iPSC-derived brain cell types. Neuron.

[164] Hammond TR, Dufort C, Dissing-Olesen L, Giera S, Young A, Wysoker A (2019). Single-cell RNA sequencing of microglia throughout the mouse lifespan and in the injured brain reveals complex cell-state changes. Immunity.

[165] Ginhoux F, Greter M, Leboeuf M, Nandi S, See P, Gokhan S (2010). Fate mapping analysis reveals that adult microglia derive from primitive macrophages. Science.

[166] Potente M, Makinen T (2017). Vascular heterogeneity and specialization in development and disease. Nat Rev Mol Cell Biol.

[167] Amann RI, Baichoo S, Blencowe BJ, Bork P, Borodovsky M, Brooksbank C (2019). Toward unrestricted use of public genomic data. Science.

[168] Penney J, Ralvenius WT, Tsai LH. Modeling Alzheimer’s disease with iPSC-derived brain cells. Mol Psychiatry. 2020;25:148–67. 10.1038/s41380-019-0468-3 PMC6906186 31391546

[169] Meyer K, Feldman HM, Lu T, Drake D, Lim ET, Ling KH (2019). REST and neural gene network dysregulation in iPSC models of Alzheimer’s disease. Cell Rep.

[170] Eisenberg E, Levanon EY (2013). Human housekeeping genes, revisited. Trends Genet.

[171] Berchtold NC, Cribbs DH, Coleman PD, Rogers J, Head E, Kim R (2008). Gene expression changes in the course of normal brain aging are sexually dimorphic. Proc Natl Acad Sci USA.

[172] Marttinen M, Paananen J, Neme A, Mitra V, Takalo M, Natunen T (2019). A multiomic approach to characterize the temporal sequence in Alzheimer’s disease-related pathology. Neurobiol Dis.

[173] Friedman B, Hansen D. Alzheimer’s gene expression by cell type—SFG. 2019. https://www.ncbi.nlm.nih.gov/geo/query/acc.cgi?acc=GSE125050.

[174] Simpson JE, Ince PG, Shaw PJ, Heath PR, Raman R, Garwood CJ (2011). Microarray analysis of the astrocyte transcriptome in the aging brain: relationship to Alzheimer’s pathology and APOE genotype. Neurobiol Aging.

[175] Xu PT, Li YJ, Qin XJ, Kroner C, Green-Odlum A, Xu H (2007). A SAGE study of apolipoprotein E3/3, E3/4 and E4/4 allele-specific gene expression in hippocampus in Alzheimer disease. Mol Cell Neurosci.

[176] Karkkainen MJ, Ferrell RE, Lawrence EC, Kimak MA, Levinson KL, McTigue MA (2000). Missense mutations interfere with VEGFR-3 signalling in primary lymphoedema. Nat Genet.

[177] Bazigou E, Lyons OT, Smith A, Venn GE, Cope C, Brown NA (2011). Genes regulating lymphangiogenesis control venous valve formation and maintenance in mice. J Clin Investig.

[178] Castorena-Gonzalez JA, Zawieja SD, Li M, Srinivasan RS, Simon AM, de Wit C (2018). Mechanisms of connexin-related lymphedema. Circ Res.

[179] Munger SJ, Davis MJ, Simon AM (2017). Defective lymphatic valve development and chylothorax in mice with a lymphatic-specific deletion of Connexin43. Dev Biol.

[180] Bazigou E, Xie S, Chen C, Weston A, Miura N, Sorokin L (2009). Integrin-alpha9 is required for fibronectin matrix assembly during lymphatic valve morphogenesis. Dev Cell.

[181] Sakai H, Fuse Y, Yamamoto T (2018). Lymphatic vessel diameter and lymphosclerosis: two different characteristics. Lymphat Res Biol.

[182] Mihara M, Hara H, Kawakami Y, Zhou HP, Tange S, Kikuchi K (2018). Site specific evaluation of lymphatic vessel sclerosis in lower limb lymphedema patients. Lymphat Res Biol.

[183] Dupont G, Iwanaga J, Yilmaz E, Tubbs RS. Connections between amyloid beta and the meningeal lymphatics as a possible route for clearance and therapeutics. Lymphat Res Biol. 2020;18:2–6. 10.1089/lrb.2018.0079 31433264

[184] Pappolla M, Sambamurti K, Vidal R, Pacheco-Quinto J, Poeggeler B, Matsubara E (2014). Evidence for lymphatic Aβ clearance in Alzheimer’s transgenic mice. Neurobiol Dis.

[185] Patel TK, Habimana-Griffin L, Gao X, Xu B, Achilefu S, Alitalo K (2019). Dural lymphatics regulate clearance of extracellular tau from the CNS. Mol Neurodegener.

[186] Wang L, Zhang Y, Zhao Y, Marshall C, Wu T, Xiao M (2019). Deep cervical lymph node ligation aggravates AD-like pathology of APP/PS1 mice. Brain Pathol.

[187] Wen Y-R, Yang J-H, Wang X, Yao Z-B (2018). Induced dural lymphangiogenesis facilities soluble amyloid-beta clearance from brain in a transgenic mouse model of Alzheimer’s disease. Neural Regen Res.

[188] Yamamoto T, Yamamoto N, Yoshimatsu H, Narushima M, Koshima I (2017). Factors associated with lymphosclerosis: an analysis on 962 lymphatic vessels. Plast Reconstr Surg.

[189] Rutkowski JM, Moya M, Johannes J, Goldman J, Swartz MA (2006). Secondary lymphedema in the mouse tail: lymphatic hyperplasia, VEGF-C upregulation, and the protective role of MMP-9. Microvasc Res.

[190] Gousopoulos E, Proulx ST, Scholl J, Uecker M, Detmar M (2016). Prominent lymphatic vessel hyperplasia with progressive dysfunction and distinct immune cell infiltration in lymphedema. Am J Pathol.

[191] Louveau A, Herz J, Alme MN, Salvador AF, Dong MQ, Viar KE (2018). CNS lymphatic drainage and neuroinflammation are regulated by meningeal lymphatic vasculature. Nat Neurosci.

[192] Hsu M, Rayasam A, Kijak JA, Choi YH, Harding JS, Marcus SA (2019). Neuroinflammation-induced lymphangiogenesis near the cribriform plate contributes to drainage of CNS-derived antigens and immune cells. Nat Commun.

[193] Schindler SE, Li Y, Todd KW, Herries EM, Henson RL, Gray JD (2019). Emerging cerebrospinal fluid biomarkers in autosomal dominant Alzheimer’s disease. Alzheimer’s Dement.

[194] Sutphen CL, McCue L, Herries EM, Xiong C, Ladenson JH, Holtzman DM (2018). Longitudinal decreases in multiple cerebrospinal fluid biomarkers of neuronal injury in symptomatic late onset Alzheimer’s disease. Alzheimer’s Dement.

[195] Taipa R, das Neves SP, Sousa AL, Fernandes J, Pinto C, Correia AP (2019). Proinflammatory and anti-inflammatory cytokines in the CSF of patients with Alzheimer’s disease and their correlation with cognitive decline. Neurobiol Aging.

[196] Krance SH, Wu C-Y, Zou Y, Mao H, Toufighi S, He X, et al. The complement cascade in Alzheimer’s disease: a systematic review and meta-analysis. Mol Psychiatry. 2019. 10.1038/s41380-41019-40536-41388. 10.1038/s41380-019-0536-8 31628417

[197] Liddelow SA, Guttenplan KA, Clarke LE, Bennett FC, Bohlen CJ, Schirmer L (2017). Neurotoxic reactive astrocytes are induced by activated microglia. Nature.

[198] Keren-Shaul H, Spinrad A, Weiner A, Matcovitch-Natan O, Dvir-Szternfeld R, Ulland TK (2017). A unique microglia type associated with restricting development of Alzheimer’s disease. Cell.

[199] Hopperton KE, Mohammad D, Trepanier MO, Giuliano V, Bazinet RP (2018). Markers of microglia in post-mortem brain samples from patients with Alzheimer’s disease: a systematic review. Mol Psychiatry.

[200] Felsky D, Roostaei T, Nho K, Risacher SL, Bradshaw EM, Petyuk V (2019). Neuropathological correlates and genetic architecture of microglial activation in elderly human brain. Nat Commun.

[201] Shi Y, Manis M, Long J, Wang K, Sullivan PM, Remolina Serrano J (2019). Microglia drive APOE-dependent neurodegeneration in a tauopathy mouse model. J Exp Med.

[202] Salomon-Zimri S, Glat MJ, Barhum Y, Luz I, Boehm-Cagan A, Liraz O (2016). Reversal of ApoE4-driven brain pathology by vascular endothelial growth factor treatment. J Alzheimer’s Dis.

[203] Claussnitzer M, Dankel SN, Kim KH, Quon G, Meuleman W, Haugen C (2015). FTO obesity variant circuitry and adipocyte browning in humans. N Engl J Med.

[204] Thul PJ, Akesson L, Wiking M, Mahdessian D, Geladaki A, Ait Blal H, et al. A subcellular map of the human proteome. Science. 2017;356:eaal3321. 10.1126/science.aal3321 28495876

[205] Janelidze S, Zetterberg H, Mattsson N, Palmqvist S, Vanderstichele H, Lindberg O (2016). CSF Abeta42/Abeta40 and Abeta42/Abeta38 ratios: better diagnostic markers of Alzheimer disease. Ann Clin Transl Neurol.

[206] Mulder C, Verwey NA, van der Flier WM, Bouwman FH, Kok A, van Elk EJ (2010). Amyloid-beta(1-42), total tau, and phosphorylated tau as cerebrospinal fluid biomarkers for the diagnosis of Alzheimer disease. Clin Chem.

[207] Bos I, Vos S, Verhey F, Scheltens P, Teunissen C, Engelborghs S (2019). Cerebrospinal fluid biomarkers of neurodegeneration, synaptic integrity, and astroglial activation across the clinical Alzheimer’s disease spectrum. Alzheimer’s Dement.

[208] Vergallo A, Megret L, Lista S, Cavedo E, Zetterberg H, Blennow K (2019). Plasma amyloid beta 40/42 ratio predicts cerebral amyloidosis in cognitively normal individuals at risk for Alzheimer’s disease. Alzheimer’s Dement.

[209] Mielke MM, Hagen CE, Xu J, Chai X, Vemuri P, Lowe VJ (2018). Plasma phospho-tau181 increases with Alzheimer’s disease clinical severity and is associated with tau- and amyloid-positron emission tomography. Alzheimer’s Dement.

[210] Mattsson N, Insel PS, Palmqvist S, Portelius E, Zetterberg H, Weiner M (2016). Cerebrospinal fluid tau, neurogranin, and neurofilament light in Alzheimer’s disease. EMBO Mol Med.

[211] Varma VR, Varma S, An Y, Hohman TJ, Seddighi S, Casanova R (2017). Alpha-2 macroglobulin in Alzheimer’s disease: a marker of neuronal injury through the RCAN1 pathway. Mol Psychiatry.

[212] Feng W, Liu HK, Kawauchi D (2018). CRISPR-engineered genome editing for the next generation neurological disease modeling. Prog Neuro-Psychopharmacol Biol Psychiatry.

[213] Zhang Y, Ulvmar MH, Stanczuk L, Martinez-Corral I, Frye M, Alitalo K (2018). Heterogeneity in VEGFR3 levels drives lymphatic vessel hyperplasia through cell-autonomous and non-cell-autonomous mechanisms. Nat Commun.

[214] Gashev AA (2002). Physiologic aspects of lymphatic contractile function: current perspectives. Ann N Y Acad Sci.

[215] Ioannou MS, Jackson J, Sheu SH, Chang CL, Weigel AV, Liu H (2019). Neuron-astrocyte metabolic coupling protects against activity-induced fatty acid toxicity. Cell.

[216] Mathys H, Davila-Velderrain J, Peng Z, Gao F, Mohammadi S, Young JZ (2019). Single-cell transcriptomic analysis of Alzheimer’s disease. Nature.

[217] Mastorakos P, McGavern D. The anatomy and immunology of vasculature in the central nervous system. Sci Immunol. 2019;4:eaav0492. 10.1126/sciimmunol.aav0492 PMC6816468 31300479

[218] Cai R, Pan C, Ghasemigharagoz A, Todorov MI, Förstera B, Zhao S (2019). Panoptic imaging of transparent mice reveals whole-body neuronal projections and skull–meninges connections. Nat Neurosci.

[219] Mahoney ER, Dumitrescu L, Moore AM, Cambronero FE, De Jager PL, Koran MEI, et al. Brain expression of the vascular endothelial growth factor gene family in cognitive aging and alzheimer’s disease. Mol Psychiatry. 2019. 10.1038/s41380-019-0458-5. [Online ahead of print]. 10.1038/s41380-019-0458-5 PMC6980445 31332262

[220] Psaltopoulou T, Sergentanis TN, Panagiotakos DB, Sergentanis IN, Kosti R, Scarmeas N (2013). Mediterranean diet, stroke, cognitive impairment, and depression: a meta-analysis. Ann Neurol.

[221] Arendt T, Stieler J, Ueberham U (2017). Is sporadic Alzheimer’s disease a developmental disorder?. J Neurochem.

[222] Hachinski V, Einhaupl K, Ganten D, Alladi S, Brayne C, Stephan BCM (2019). Preventing dementia by preventing stroke: the Berlin manifesto. Alzheimer’s Dement.

[223] Mestre H, Du T, Sweeney AM, Liu G, Samson AJ, Peng W (2020). Cerebrospinal fluid influx drives acute ischemic tissue swelling. Science.

[224] Costerus JM, Brouwer MC, van de Beek D (2018). Technological advances and changing indications for lumbar puncture in neurological disorders. Lancet Neurol.

[225] Mohammad MG, Tsai VWW, Ruitenberg MJ, Hassanpour M, Li H, Hart PH (2014). Immune cell trafficking from the brain maintains CNS immune tolerance. J Clin Investig.

[226] Mentis AA, Pantelidi K, Dardiotis E, Hadjigeorgiou GM, Petinaki E (2018). Precision medicine and global health: the good, the bad, and the ugly. Front Med.

[227] Kontou PI, Pavlopoulou A, Bagos PG (2018). Methods of analysis and meta-analysis for identifying differentially expressed genes. Methods Mol Biol.

[228] Bolstad BM, Collin F, Brettschneider J, Simpson K, Cope L, Irizarry R, et al. Quality assessment of Affymetrix GeneChip data. In: Bioinformatics and computational biology solutions using R and bioconductor. Springer; Berlin/Heidelberg, Germany; 2005. p 33–47.

[229] Benjamini Y, Hochberg Y (1995). Controlling the false discovery rate: a practical and powerful approach to multiple testing. J R Stat Soc.

[230] Robinson MD, McCarthy DJ, Smyth GK (2010). edgeR: a bioconductor package for differential expression analysis of digital gene expression data. Bioinformatics.

[231] McCarthy DJ, Chen Y, Smyth GK (2012). Differential expression analysis of multifactor RNA-Seq experiments with respect to biological variation. Nucleic Acids Res.

[232] Mostafavi S, Gaiteri C, Sullivan SE, White CC, Tasaki S, Xu J (2018). A molecular network of the aging human brain provides insights into the pathology and cognitive decline of Alzheimer’s disease. Nat Neurosci.

[233] Gotz J, Bodea LG, Goedert M (2018). Rodent models for Alzheimer disease. Nat Rev Neurosci.

[234] Verheijen J, Sleegers K (2018). Understanding Alzheimer disease at the interface between genetics and transcriptomics. Trends Genet.

[235] Lanoiselee HM, Nicolas G, Wallon D, Rovelet-Lecrux A, Lacour M, Rousseau S (2017). APP, PSEN1, and PSEN2 mutations in early-onset Alzheimer disease: a genetic screening study of familial and sporadic cases. PLoS Med.

[236] Winchester LM, Powell J, Lovestone S, Nevado-Holgado AJ (2018). Red blood cell indices and anaemia as causative factors for cognitive function deficits and for Alzheimer’s disease. Genome Med.

[237] Kunkle BW, Grenier-Boley B, Sims R, Bis JC, Damotte V, Naj AC (2019). Genetic meta-analysis of diagnosed Alzheimer’s disease identifies new risk loci and implicates Abeta, tau, immunity and lipid processing. Nat Genet.

[238] Ojelade SA, Lee TV, Giagtzoglou N, Yu L, Ugur B, Li Y (2019). cindr, the Drosophila homolog of the CD2AP Alzheimer’s disease risk gene, is required for synaptic transmission and proteostasis. Cell Rep.

[239] Jansen IE, Savage JE, Watanabe K, Bryois J, Williams DM, Steinberg S (2019). Genome-wide meta-analysis identifies new loci and functional pathways influencing Alzheimer’s disease risk. Nat Genet.

[240] Jang H, Bae JB, Dardiotis E, Scarmeas N, Sachdev PS, Lipnicki DM (2018). Differential effects of completed and incomplete pregnancies on the risk of Alzheimer disease. Neurology.

[241] Anastasiou CA, Yannakoulia M, Kosmidis MH, Dardiotis E, Hadjigeorgiou GM, Sakka P (2017). Mediterranean diet and cognitive health: initial results from the Hellenic Longitudinal Investigation of Ageing and Diet. PloS ONE.

[242] Servick K. Another major drug candidate targeting the brain plaques of Alzheimer’s disease has failed. What’s left. Science. 2019;10. https://www.sciencemag.org/news/2019/03/another-major-drug-candidate-targeting-brain-plaques-alzheimer-s-disease-has-failed (Accessed 6 August 2019).

[243] Park JS, Lee J, Jung ES, Kim MH, Kim IB, Son H (2019). Brain somatic mutations observed in Alzheimer’s disease associated with aging and dysregulation of tau phosphorylation. Nat Commun.

[244] Wang Z, Lei H, Zheng M, Li Y, Cui Y, Hao F (2016). Meta-analysis of the association between Alzheimer disease and variants in GAB2, PICALM, and SORL1. Mol Neurobiol.

[245] Wingo TS, Cutler DJ, Wingo AP, Le N-A, Rabinovici GD, Miller BL, et al. Association of early-onset Alzheimer disease with elevated low-density lipoprotein cholesterol levels and rare genetic coding variants of APOB. JAMA Neurol. 2019;76:809–17. 10.1001/jamaneurol.2019.0648 PMC6547122 31135820

[246] Bis JC, Jian X, Kunkle BW, Chen Y, Hamilton-Nelson KL, Bush WS, et al. Whole exome sequencing study identifies novel rare and common Alzheimer’s-Associated variants involved in immune response and transcriptional regulation. Mol Psychiatry. 2018. 10.1038/s41380-018-0112-7. 10.1038/s41380-018-0112-7 PMC6375806 30108311

[247] Ayton S, Wang Y, Diouf I, Schneider JA, Brockman J, Morris MC, et al. Brain iron is associated with accelerated cognitive decline in people with Alzheimer pathology. Mol Psychiatry. 2019. 10.1038/s41380-019-0375-7. [Online ahead of print]. 10.1038/s41380-019-0375-7 PMC6698435 30778133

[248] Goozee K, Chatterjee P, James I, Shen K, Sohrabi HR, Asih PR, et al. Elevated plasma ferritin in elderly individuals with high neocortical amyloid-β load. Mol Psychiatry. 2018;23:1807–12. 10.1038/mp.2017.146 28696433

[249] Zhang S, Cai F, Wu Y, Bozorgmehr T, Wang Z, Zhang S, et al. A presenilin-1 mutation causes Alzheimer disease without affecting Notch signaling. Mol Psychiatry. 2020;25:603–13. 10.1038/s41380-018-0101-x 29915376

[250] Hartl D, May P, Gu W, Mayhaus M, Pichler S, Spaniol C, et al. A rare loss-of-function variant of ADAM17 is associated with late-onset familial Alzheimer disease. Mol Psychiatry. 2020;25:629–39. 10.1038/s41380-018-0091-8 PMC7042727 29988083

[251] Roussos P, Katsel P, Fam P, Tan W, Purohit DP, Haroutunian V (2015). The triggering receptor expressed on myeloid cells 2 (TREM2) is associated with enhanced inflammation, neuropathological lesions and increased risk for Alzheimer’s. Dement Alzheimer’s Dement.

[252] Deming Y, Filipello F, Cignarella F, Cantoni C, Hsu S, Mikesell R, et al. The MS4A gene cluster is a key modulator of soluble TREM2 and Alzheimer’s disease risk. Sci Transl Med. 2019;11:eaau2291. 10.1126/scitranslmed.aau2291 PMC6697053 31413141

[253] Maloney B, Lahiri DK (2016). Epigenetics of dementia: understanding the disease as a transformation rather than a state. Lancet Neurol.

[254] Wolters FJ, Zonneveld HI, Licher S, Cremers LGM, Ikram MK, Koudstaal PJ, et al. Hemoglobin and anemia in relation to dementia risk and accompanying changes on brain MRI. Neurology. 2019;93:e917–26. 10.1212/WNL.0000000000008003 PMC6745727 31366722

[255] Peters ME, Schwartz S, Han D, Rabins PV, Steinberg M, Tschanz JT (2015). Neuropsychiatric symptoms as predictors of progression to severe Alzheimer’s dementia and death: the Cache County Dementia Progression Study. Am J Psychiatry.

[256] Burhanullah MH, Tschanz JT, Peters ME, Leoutsakos JM, Matyi J, Lyketsos CG, et al. Neuropsychiatric symptoms as risk factors for cognitive decline in clinically normal older adults: the Cache County Study. Am J Geriatric Psychiatry. 2020;28:64–71. 10.1016/j.jagp.2019.03.023 PMC6874723 31186157

[257] Buttler K, Lohrberg M, Gross G, Weich HA, Wilting J (2016). Integration of CD45-positive leukocytes into newly forming lymphatics of adult mice. Histochemistry Cell Biol.

[258] Kajiwara Y, Wang E, Wang M, Sin WC, Brennand KJ, Schadt E (2018). GJA1 (connexin43) is a key regulator of Alzheimer’s disease pathogenesis. Acta Neuropathol Commun.

[259] Stern RA, Adler CH, Chen K, Navitsky M, Luo J, Dodick DW (2019). Tau positron-emission tomography in former national football league players. N Engl J Med.

[260] Sabine A, Agalarov Y, Maby-El Hajjami H, Jaquet M, Hagerling R, Pollmann C (2012). Mechanotransduction, PROX1, and FOXC2 cooperate to control connexin37 and calcineurin during lymphatic-valve formation. Dev Cell.

[261] Zheng W, Aspelund A, Alitalo K (2014). Lymphangiogenic factors, mechanisms, and applications. J Clin Investig.

[262] van Steensel MA, Damstra RJ, Heitink MV, Bladergroen RS, Veraart J, Steijlen PM (2009). Novel missense mutations in the FOXC2 gene alter transcriptional activity. Hum Mutat.

[263] Alders M, Hogan BM, Gjini E, Salehi F, Al-Gazali L, Hennekam EA (2009). Mutations in CCBE1 cause generalized lymph vessel dysplasia in humans. Nat Genet.

[264] Gordon K, Schulte D, Brice G, Simpson MA, Roukens MG, van Impel A (2013). Mutation in vascular endothelial growth factor-C, a ligand for vascular endothelial growth factor receptor-3, is associated with autosomal dominant milroy-like primary lymphedema. Circ Res.

[265] Ferrell RE, Baty CJ, Kimak MA, Karlsson JM, Lawrence EC, Franke-Snyder M (2010). GJC2 missense mutations cause human lymphedema. Am J Hum Genet.

