# Supplementary material for: Apolipoprotein E4 and meningeal lymphatics in Alzheimer disease: a conceptual framework
Source: Mol Psychiatry. 2020 Apr 30;26(4):1075–97. doi: 10.1038/s41380-020-0731-7 (PMC7985019; doi:10.1038/s41380-020-0731-7)
Supplement: Supplementary file 9 — Supplementary Figures and Tables Legends [file 41380_2020_731_MOESM9_ESM.docx]

**SUPPLEMENTARY MATERIAL**

**Supplementary File 1.** Excel table with the studies identified by systematic query, their GEO accession number, the decision on their inclusion or exclusion from being reanalyzed (and the accompanying rationale), and the number of samples per each study **(XLSX format).**

**Supplementary File 2.** Excel table with characteristics of studies submitted to re-analysis **(XLSX format).**

**Supplementary File 3.** Excel table with cell counts’ numbers of the single-cell next-generation sequencing study by Mathys *et al.* (2019) reanalyzed **(XLSX format).**

**Supplementary Figure 1.** Box and Whisker plot showing gene expression levels in the study with GEO accession number “GSE48350” **(PDF format).**

**Supplementary Figure 2.** Box and Whisker plot showing gene expression levels in the study with GEO accession number “GSE106241” **(PDF format).**

**Supplementary Figure 3.** Box and Whisker plot showing gene expression levels in the study with GEO accession number “GSE125050” **(PDF format).**

**Supplementary Figure 4.** Box and Whisker plot showing gene expression levels in the study with GEO accession number “GSE29652” **(PDF format).**

**Supplementary Figure 5.** Scatter plot showing gene expression levels in the study with GEO accession number “GSE6677” **(PDF format).**
